# The war on deciduous forest: Large-scale herbicide treatment in the Swedish boreal forest 1948 to 1984

**DOI:** 10.1007/s13280-021-01660-5

**Published:** 2021-11-16

**Authors:** Lars Östlund, Sandra Laestander, Gerd Aurell, Greger Hörnberg

**Affiliations:** 1 Department of Forest Ecology and Management, Swedish University of Agricultural Sciences, Umeå, Sweden; 2 Present Address: Sveaskog AB, Arvidsjaur, Sweden; 3 Department of Forest Ecology and Management, Swedish University of Agricultural Sciences, Umeå, Sweden; 4 Umeå Academy of Fine Arts, Umeå University, Umeå, Sweden; 5 Present Address: County Board Jämtland, Östersund, Sweden

**Keywords:** 2,4-D, 2,4,5-T, Birch forest, Environmental protests, Forest history, Herbicides, Hormoslyr

## Abstract

At the mid-twentieth century the pace of the transformation of the Swedish forest increased. New methods; large-scale clearcutting, mechanization of logging and planting of seedlings were developed. Chemicals were used to control insects and unwanted tree species. The aims of this study were to elucidate the timing, chain of events and the spatial extent of the large-scale spraying of phenoxy acids in Swedish forests and the drivers for this practice. More than 700 000 hectares of productive forest land was sprayed and the main driving force was a strong will to transform the forest into high-yield coniferous forest plantations. We conclude that; (1) the use of herbicides in forestry in Sweden was done on a very large scale in the period 1948–1984, (2) the ecosystem legacy of herbicide spraying must be investigated and (3) a homogenous cadre of like-minded professionals working across commercial companies, state agencies and universities is dangerous.

**Supplementary Information:**

The online version contains supplementary material available at 10.1007/s13280-021-01660-5.

## Introduction

During the last 200 years, forest resources from the periphery have successively been drawn into the rapidly growing economies of Western Europe (Williams 1992; Pollard 1997). The industrial revolution, starting in the eighteenth century, created a strong demand for wood products, which could only be supplied by regions that had not already depleted or reduced their forests for agricultural and other land uses (Perlin 1991). The northern parts of Scandinavia were heavily forested at that time, and this old-growth forest was therefore suddenly interesting for the industrially developing countries and integrated into the world economy. Before this shift, the main anthropogenic impacts on the forest were localized harvests of wood for fuel and construction (Rautio et al. 2016) together with use of fire to create more suitable forest landscapes for grazing (Niklasson and Granström 2000; Hörnberg et al. 2018). In pre-industrial time in the early nineteenth century the overall exploitation of the forest was very limited and the population density remained low (< 1 person/km^2^) so the forest landscapes were still primarily structured by non-anthropogenic forces (Östlund et al. 1997; Rautio et al. 2016).

The transformation of the northern forest in Scandinavia since the pre-industrial period until present time has, for the most part, been a gradual process where added uses—agrarian, pre-industrial and industrial—have resulted in a successively increased pressure on the forest. The overall theme in this process has been a shift from ancient low-intense agrarian and indigenous Sami pastoral reindeer herding towards the present broad scale and high intensive industrial forest use.

Although the main changes have been gradual and aligned with much broader socio-economic processes there have also been some very rapid developments. One of these is the high-grading of the northern old-growth Scandinavian Scots pine (*Pinus sylvestris* L.) forest in the late nineteenth century (Björklund 1984; Östlund et al. 1997; Linder and Östlund 1998). In this process, very old Scots pines (250 to more than 600 years old at the time), were logged across the interior of northern Sweden and almost all inland forests were affected. A similar timber-frontier moved across northern Finland, northern Russia (Björklund 1984) and the North American continent at the same time (Williams 1992). The removal of millions of ancient trees across these regions had massive effects on the boreal forest ecosystems, including loss of key ecological features of the forest and was the start of the transformation towards production forest. The late 1800s was a period of large-scale forest exploitation in northern Sweden (Östlund et al. 1997), and the early 1900s was a period of transition towards sustainable forestry in a production sense (Lundmark 2020).

Another rapid development was the introduction of clear-cutting forestry (Lundmark 2020). This had been trialed and implemented on a limited scale even before 1900 (Lundmark et al. 2013, 2017), but was increasingly widely applied thereafter and clear-cutting became the main tree harvesting method in around 1950. Clear-cutting changed the appearance and structure of the forest landscape and, together with targeted regeneration practices, resulted in the first single-species, one-storied, single-aged forests in northern Sweden. This transition has had very complex consequences, but the most important may be the general loss of biodiversity, key structures and creation of debts in terms of both species and habitat losses (Fridman and Walheim 2000; Stenbacka et al. 2010). Unsurprisingly, these losses prompted the initiation of strong conservation movements in Sweden, together with broad advocation of less intensive forestry and consideration of values other than timber production (Simonsson et al. 2015).

The developments outlined above have been relatively well studied and both the driving forces and consequences are well known, but another very broadly applied forestry practice from recent past has received very little attention. This is the widespread application of herbicides from the end of the 1940s to the start of the 1980s to eradicate broadleaves in young forest stands (Bärring 1978; Lisberg-Jensen 2006; Laestander 2015). Two main herbicides were used, 2,4-D (dichlorophenoxyacetic acid) and 2,4,5-T (trichlorophenoxyacetic acid), both of which are phenoxy acids and acting systematically, causing uncontrolled growth and finally death of deciduous plants and trees (Bovey and Young 1980). Usually these two chemicals were used together to increase their effectiveness in a mixture known as *Hormoslyr* in Sweden, *Tormona* in central Europe and *Agent Orange* during the Vietnam war in the 1960s. The herbicides were applied in Sweden to reduce competition from unwanted deciduous trees, primarily birches (*Betula* spp.), aspens (*Populus tremula* L.), goat willow (*Salix caprea* L.) and rowans (*Sorbus aecuparia* L.) in managed forests dominated by Scots pine and Norway spruce (*Picea abies*). Herbicides were used as complementary tools to other forestry measures and were applied either by aircraft or manually, by spraying with hand-held equipment.

The primary reason for the use of herbicides was the major change in forest management methods in the 1940s and 1950s. Although clear-cutting had been trialled and applied for decades, it became the standard method for harvesting trees from the 1950s onwards (Lundmark et al. 2013). The change in management in combination with the transition from largely old-growth forests to younger production forests after the dramatic exploitation of the 1800s created new challenges. One was the natural regeneration of deciduous trees in the large clear-cuts, a management problem that was amplified by the pressure from the forest industry to provide wood from coniferous trees for the sawmills and pulpmills. This coincided with a time when grazing by domestic animals in the forest declined rapidly because people abandoned farming and also because browsing by wild animals (primarily moose, *Alces alces* L.) was very limited due to high hunting pressure for more than a century.

In Swedish forestry the first experiments on phenoxy acids (more precisely chlorophoxyacetic acids) herbicides were carried out in 1947 (Häggström 1956). The development of these herbicides came from experiments done in Great Britain and the United States during the Second World War with a purpose to produce chemicals which could be used in warfare against the food production in Germany. The most frequently used phenoxy acids in Swedish forestry were 2,4-D and 2,4,5-T (as mentioned), but substantial amounts of MCPA (2-methyl-4-chlorophenoxyacetic acid) were also applied (Anon. 1974; Bärring 1978). The phenoxy herbicides’ effectiveness varied species-dependently. 2,4-D was considered to be the most appropriate herbicide for sensitive species such as alder (*Alnus* spp. Mill), birch and goat willow, whereas 2,4,5-T was more effective against resistant species such as oak (*Quercus* spp. L.) and other temperate deciduous trees (Anon. 1974). The commercial products often consisted of a 2:1 mixture of 2,4-D and 2,4,5-T. One of the most widely known preparations, Hormoslyr 64, consisted of a mixture of 2,4-D and 2,4,5-T in ester forms. In the 2,4,5-T production some contaminants were formed, of which the dioxin 2,3,7,8-TCDD was the most toxic (Lilienfeld and Gallo 1989). The use of herbicides in forestry began in the late 1940s and was very widely practiced during the coming decades, but triggered one of the largest environmental protests ever in Sweden in the late 1970s, which resulted in a complete ban in 1984. A broader historical background is found in Supplementary Information (S1).

The motivation for this study is to investigate a period in Swedish forestry which is today largely unknown and which had a fundamental impact on the forestry sector at the end of the twentieth century and reaching into present time. The overall aims of the study presented here were to give a broad narrative of the timing and chain of events leading to the large-scale herbicide spraying in the Swedish boreo-nemoral and boreal forests, as well as the main socio-economic and cultural drivers that initiated, sustained and eventually ended the practice. The following questions guided the investigation.

- When and how did the herbicide treatment of forests in northern Sweden begin, and what were the main drivers initially?
- How did the practice develop, and what types of forests were sprayed?
- How extensive was herbicide treatment nationally during the studied period?
- How extensive was herbicide treatment in a regional perspective during the studied period?
- When and how did opposition to herbicide treatment begin and how did it affect the practice?
- Why did herbicide treatment of the forest in northern Sweden stop?

We have addressed these questions using primary data collected mainly from archives of the principal actors: the forestry companies using the herbicides. Here, the data are applied to provide an inside story regarding the companies’ practices and briefly compared to limited available national and international statistics. To get a broader understanding we have then also contrasted this story to narratives of those who were engaged in the use of or protested against the herbicides in forestry. Consequences of the ‘herbicide-period’ in Swedish forestry are also discussed in terms of ecological changes to the forest ecosystems and human aspects from both domestic and international perspectives.

## Materials and methods

### Study area

The primary study area comprises roughly the northern two-thirds of Sweden, encompassing 242 735 km^2^, and lies within the boreal and northernmost boreo-nemoral part of Sweden (based on locations of the studied forest companies), but we also attempt to approximate the total area sprayed with herbicides in Sweden (Fig. S11). The main tree species in the region include (in order of dominance): Scots pine, Norway spruce, downy birch (*Betula pubescens*), silver birch (*Betula pendula*), trembling aspen, goat willow and rowan. The conifers, pine and spruce, are by far the most abundant, accounting now and historically for approximately 80% of the growing stock of timber in boreal Sweden (Forestry Statistics of Sweden 2021).

### Historical records from archives

This study is primarily based on analysis of historical records from archives of four commercial forest companies in Sweden, which are known to have extensively used herbicides: Domänverket (the Swedish National forest company, today Sveaskog AB and Fastighetsverket), Svenska Cellulosa Aktiebolaget (SCA), Stora Kopparberg AB and Uddeholmsbolaget. We also searched for relevant historical records in archives of Billerud-Korsnäs AB and Kopparfors AB with no success (mainly due to lack of organization of vast historical material that is not openly available for research). We have also used published data from Modo AB (Andrén 1992). A large share (> 40%) of the productive forest land in the studied region was (and is) owned by forest companies. Collectively, their holdings of forest land amounted to approximately seven million hectares (ha) during the studied time period. There were some shifts in land-ownership during this period, but we consider them of minor importance for the overall results. Other commercial forest companies, private landowners and forest commoners also used herbicides during this period, but finding and systematically compiling all relevant archival material would not have been feasible, so we largely limited our study to the abovementioned commercial forest companies.

Most of the documents are unsorted or coarsely sorted into different folders in the archives, so to extract and interpret relevant information we sorted the documents into the following three categories. First, data on the extent of herbicide spraying between 1945 and 1984 by each company. Second, general documentation of herbicide use (forest management plans, methods of application, results of herbicide applications, and other technical aspects). This type of primary historical records have been used in many studies of forestry and forest management in Sweden and abroad (Östlund et al 1997; Buergi and Schuler 2003; Boucher et al 2009). Such records are reliable historical sources since they were produced internally to facilitate the management of the forests and thus needed to be precise and accurate. There is therefore no reason to believe that the existing records have been manipulated in any way. The main problem when it comes to the archival material from the forest companies is that some material may have been destroyed either deliberately or by accident, or is registered in such a way that it is not possible to extract today.

A third category of records related to herbicide use was also found in the archives. These records was mostly connected to outreach to the public, including saved newspaper articles. These records are of a very different kind in relation to the ones mentioned above. They are biased and express the opinions and propaganda from each forest company, or in some cases relates the conflicts between environmentalists and the forest companies.

All the archival sources are noted in the Supplementary Information (S10) and each specific source material used in the results is referred to by a specific number; for example S10:1.

### Interviews

We also interviewed three retired foresters, two retired forest workers and six environmental activists who protested against the herbicide treatments, to put the archival data into context and improve understanding of the driving forces of the use and of the protests as well as the technicalities of practical herbicide use. We conducted the (semi-structured) interviews (cf. Jacob and Furgerson 2012), with pre-prepared open questions, then recorded and subsequently transcribed the interviews. The questions we asked focused on the practical work with herbicides, the management of herbicide spraying, the discussion within the forest companies and the local environmental groups, the start of the protests, the reactions from the forest companies etc. The questions to the different categories of people that we interviewed are listed in S12.

## Results

### The introduction and early practice of herbicide treatment in Sweden

**Fig. 1:**
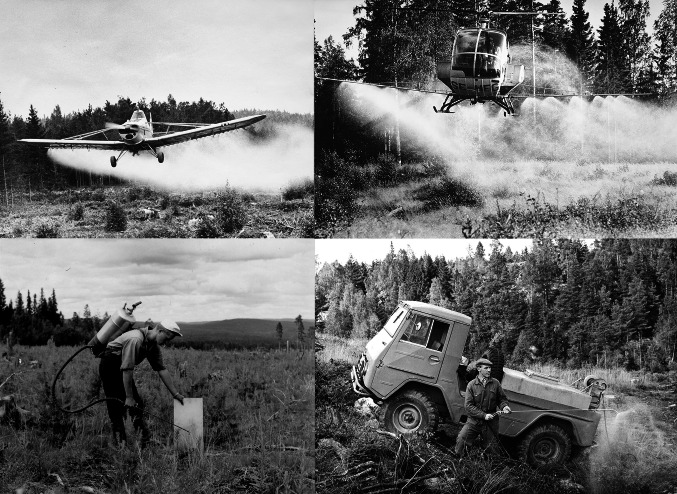
The different methods for applying herbicides; airplanes, helicopters, manual spraying and the use of a terrain vehicles. Upper photos by Leif Öster (Domänverket photoarchive at riksarkivet Härnösand); Lower photos at Skogsbibliotekets fotoarkiv, SLU, Umeå

The forest companies began their herbicide treatments in practical field-trials, sometimes in collaboration with producers of the herbicides and sometimes in collaboration with the Forest Research Institute (Sw. Statens Skogsförsöksanstalt) (S10:23). The archive material shows that various application methods were used, but mainly they were applied in notches in trees’ trunks or via manual spraying by workers carrying containers on their back (S10:4,23) (Fig. 1) (Fig. S5a–b). The major initial concerns were the selection of suitable sites to spray, the dosage of herbicides per hectares and the most effective mixture of herbicides for targeted species (S10:1,7)). The state forest company Domänverket and SCA focused on recent clear-cuts and areas where deciduous trees had re-sprouted after manual clearing with axes in the northernmost parts of their holdings. In these areas, the natural regeneration of deciduous trees became problematic when pure stands of Scots pine and Norway spruce were desired (S10:4). The first field trials showed that trees up to 3 m tall and birch shoots from stumps could be effectively killed with the herbicides. Success rates of 60–90% (percentage of deciduous trees killed) were reported (S10:23). Typical sizes of areas where herbicides were tried varied from a few to around 50 hectares in this early stage (S10:7). However, in some districts large-scale treatments began almost directly, e.g., in Domänverket’s holdings in the Arjeplog district (S10:10). The main issue discussed during this period was the cost of herbicide treatment versus manual clearing of deciduous trees on clear-cuts (S10:4,23).

At the mid-1950s larger-scale and cheaper methods were employed more extensively. Both helicopters and airplanes were used to spray larger areas (S10:1,10; Fig. S6). The feasibility and effectiveness of using hand-held but motorized spraying units to treat areas that were too small for helicopters were also experimentally assessed (S10:23). Another discussion was also whether to use water or diesel as the carrier for the herbicide. Diesel was found to be more effective, since it made the chemicals stick to the trees, but on the other hand was more costly. Thus both methods were used in parallel (Bill Wahlgren oral communication). The size of sprayed stands also grew during this time, sometimes up to hundreds of hectares (S10:4) and also included older deciduous forests (Fig. S8). The transition was very rapid and included collaboration on all levels of the companies’ forest management departments (S10:23) and a very close network of forest companies, chemical companies and specialized entrepreneurs developed. The specialist company Basbolaget played a particularly important role in the aerial spraying, including training pilots and transporting the herbicides to temporary airfields near the sites to be sprayed. Basbolaget employed foresters and also gave very explicit instructions to the forest companies about arrangements before spraying (S10:8,9,23). The chemical manufacturing company Gullviks Fabriks AB also provided airplanes and logistical support for spraying sites (S10:23).

During this period, herbicides were used much more extensively in some regions than in others, especially in the state forests (S10:1). Up until around 1960 there is hardly any evidence of any internal discussions regarding either human health or environmental concerns linked to the use of herbicides. Contrarily, there are many indications that the foresters were pleased with the development of a new and very powerful solution for a major forest management problem. This optimistic view of chemical solutions is well exemplified by a poster from Gullviks AB displaying more than 30 chemical products designed to eradicate a range of broadleaved trees and bushes, insects, birds and other vertebrates (Fig. S4).

### Sprayed area in Sweden 1950–1984

**Fig. 2:**
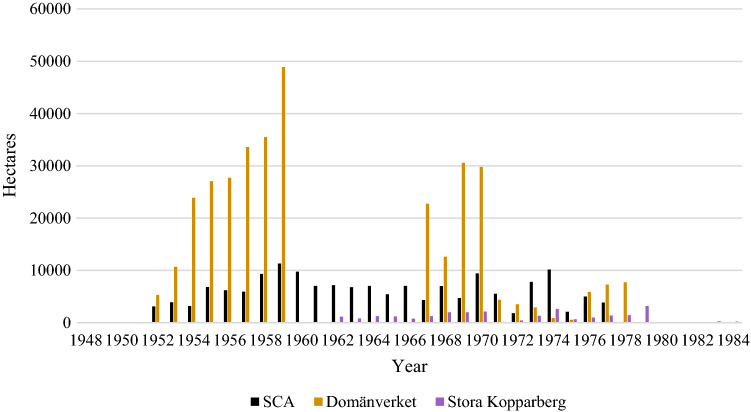
Herbicide treatment in forests (hectares of forest land) as recorded by the forest companies SCA AB, Domänverket and Stora Kopparberg AB. NB. that records are missing from 1960 to 1966 for Domänverket. (S10:1, 2, 22, 26, 27, 28, 29)

According to our data from the three studied forest companies, 545 421 ha of forest land they owned were sprayed with herbicides between 1950 and 1984 (S10:1, 22, 26, 27, 28, 29) (Fig. 2). There are gaps in this data-series, and the most important are the years 1960–1966 for Domänverket. Scattered data from districts show that this was an intensive period of herbicide use at Domänverket. Of this area, 65, 30 and 5% was owned by Domänverket, SCA AB and Stora Kopparberg AB, respectively. The intensity of herbicide use by these three forest companies was fairly similar, as they sprayed 10.0, 9.9 and ca. 7% of their total productive forest land, respectively, during this period.

An official report (Anon. 1974) presented compilations of the forest areas treated nationally with herbicides during part of the period (1968 to 1972). According to this report, the areas treated with herbicides in 1968, 1969 and 1970 amounted to 64 000, 98 000 and 92 000 ha, respectively. Thus, the total area sprayed during these three years amounted to 254 000 ha (Anon. 1974). Our compiled data indicates that the three focal companies treated 113 422 ha with herbicides, 51% of the total, during this short period. From the difference between these totals we can conclude that at least 685 999 ha of forest land was sprayed between 1950 and 1984 nationally. Moreover, if the relation between the areas sprayed by the companies (according to the records we examined) and the national total remained the same during the period 1948–1984, approximately 1.1 million ha was sprayed in Sweden during that time.

### Regional differences in herbicide spraying in northern Sweden

**Fig. 3:**
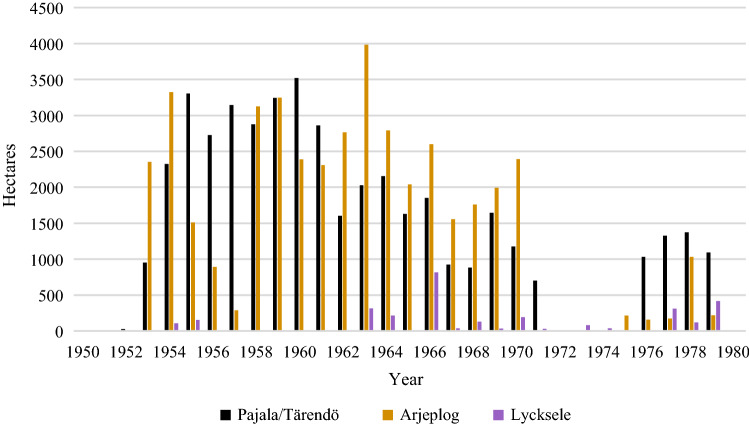
Herbicide treatment of forests (in hectares) for the districts (Sw. revir) of Pajala/Tärendö, Arjeplog and Lycksele at the forest company Domänverket (S10:8–21)

Most of the national forest land managed by Domänverket that was sprayed with herbicides, was located in the most northerly inland region of Sweden, and more than 90% was located north of the town Umeå (63° N) (S10:1). However, even in this northern region there was wide variation in use of herbicides (Fig. 3). In some districts (Sw. revir) they were only used sporadically (S10:1). In contrast, they were used much longer and more extensively in other districts in the region (particularly Arjeplog, Arvidsjaur, Skellefteå, Åsele and Torneå) and almost not at all in one district (Malå), where the well-known forester Joel Wretlind was in charge during most of the studied period.

Most of the land sprayed with herbicide by the commercial forest company SCA AB was also located in the northernmost parts of the company’s forest holdings. Like Domänverket, SCA clearly used herbicides more extensively in some districts than in others, and there was larger between-year variation in the company’s total applications. The herbicide use by Stora Kopparberg AB and Uddeholmsbolaget, both of their land holdings located more to the south, also began much later (mainly in the 1960s) compared to the companies owning land in the northern part of the boreal forest.

### Opposition to and the end of herbicide treatment in Swedish forests

In the forest companies’ archives we found no documents mentioning internal opposition or external protest against herbicide treatment before 1956, when the forester Fredrik Ebeling gave a lecture to the annual national assembly of the Swedish Society for Nature Conservation (Sw. Naturskyddsföreningen). Ebeling was a leading forester at Domänverket at the time and later became chief executive of the National Board of Forestry. His lecture was intended to explain the necessity of using herbicides in Swedish forestry based on the contemporary state of the forest and the importance of conifers for timber production. This appears to have been an early attempt to counter criticism directed from Naturskyddsföreningen, the main nature conservation NGO in Sweden (S10:4).

From the late 1960s onwards increasing conflicts are documented in the company archives. Initially these were relatively polite responses to public criticism, internal letters about how to inform the public, and material intended to educate and inform target audiences about the safety and important roles of herbicides (S10:4,5,6,7,25). Pamphlets and posters were distributed at prospective sites of herbicide treatments by the companies’ employees (S10:25). One ambitious pamphlet produced by SCA AB, based on a similar pamphlet by Domänverket, was entitled *Questions and answers regarding phenoxy acids* (Fig. S3). As suggested by the title, this pamphlet poses and answers a number of questions, including the following examples; “Are phenoxy acids poisonous?”, “Can’t deciduous trees be cleared manually instead?”, “Can you eat berries from a herbicide-sprayed clear-cut?” The answers all categorically refuted the possibility that the herbicides posed any threats, in a reasoning educational manner (S10:23). In a similar manner, a 16 mm film entitled *Chemical control of deciduous trees* (Sw. Kemisk lövbekämpning) showing how to use herbicides and their effectiveness was also produced by Domänverket (S2).

In the early 1970s more detailed instructions for dealing with the chemicals were issued (S10:4). Staff using herbicides were instructed to protect their faces with plastic shields, avoid spraying in wind and wash their hands before eating.

Domänverket’s executive officer Folke Rydbo gave a public speech about herbicides and forestry in 1971 in an effort to counter public opposition. In this speech he stated that banning herbicides would cause additional annual costs of ca. 4 million Swedish Crowns, so continued use was essential for the Swedish forestry sector’s international competitiveness (S10:4). Domänverket repeated this claim when they published calculations purporting to show the economic consequences of banning herbicides, indicating (for example) that it would incur additional net costs of 3.7 million SEK in 1972 (S10:4).

During the 1970s public protests began in various places and often included civil disobedience at sites where the forest companies planned to spray herbicides (Fig. S7a–c, S9). The archives contain many documents recording these events; complaints in letters sent to television reporters, copies of newspaper articles, documentation of meetings with protesters and communications within and between forest companies (S10:4,5,6,7). Thorough instructions were produced about how to act when environmental activists attacked forest company employees publicly (S10:5). These included detailed guidance for understanding the environmentalists’ arguments and effective ways to counter them.

In 1978 and 1979 many local protests in small towns and villages where forest companies had recently sprayed or planned to spray herbicides escalated, leading to confrontation with the forest companies and civil disobedience. Some of the places where such local opposition arose are Torsby in Värmland, Klaxåsen in Jämtland, Sarkavaara/Jokkmokk and Arjeplog in Norrbotten. One of the most well-known conflicts occurred in the village Aapua in Norrbotten, close to the Finnish border, where villagers protested against planned herbicide spraying by Domänverket in forests nearby (Fig. S7a–c). The protests were aroused by fears of the herbicides’ toxicity and losses of local employment in manual clearing in the forest, especially for women. The protests attracted news teams from local, national and even international newspapers, television stations and radio channels to the small village.

## Discussion

One of the most rapid and pervasive changes in Swedish forest management history was the broad-scale use of herbicides. It progressed from experiments in 1947 to a widespread practice for reducing deciduous components of forest ecosystems on state and private forest land during the 1950s, 1960s and 1970s. Furthermore, herbicides were widely applied with no analysis of environmental consequences on flora and fauna or effects on the health of people exposed to them through either work or for example by eating berries from areas sprayed with herbicides. In this paper we have distinguished three phases of the herbicide period in Swedish forestry.

### The beginning of herbicide use in Swedish forests

The first period of herbicide use, from the late 1940s until the mid-1950s, was characterized by experimentation with the recently discovered, very effective phenoxy acids (Fransson 1952). They were introduced at a time of transition in forestry and forest management in northern Sweden. The logged-over old-growth forest was being turned into a production forest (Östlund et al. 1997) and the large scale clear-cutting led to unwanted regeneration of deciduous trees (Axelsson et al. 2002).

One of the interviewees, retired forester Bertil Stenlund from Lycksele described this period as follows:

So what happened with these big clear cuts, because they were giant clear cuts, they could be 1000 hectares, what happened? The birch took over. And the poor seedlings of spruce and pine that we planted couldn’t compete. Then hormoslyr [the 2,4-D and 2,4,5-T herbicide mixture] came like a rescuing angel.

The chemical clearing was much cheaper than manual work and documents in the archives give the impression that a ‘novelty’ factor also played a role in the choice to use these new herbicides instead of traditional manual methods. Bertil Stenlund recalled:

The ambition was to cut the old logged-over, residual forest and make new forest grow, and this thought animated all of us. You see, the biggest star among us was not the one who delivered the most money, but the one who made the forest grow back again.

This is clearly connected to the general societal trends during the 1950s, which included strong urbanisation, modernisation of agriculture and the introduction of new methods and chemicals in everyday life. There was also clearly no consideration of health risks to workers during this period (Lisberg-Jensen 2006). In stark contrast, photos show workers spraying and handling herbicides with no protective gear. Retired forest worker Bertil Johansson in Älvsbyn remembered well this carelessness in real practice:

We had no idea what this hormoslyr [mix of 2,4-D and 2,4,5-T herbicide] was. In the forest no one really washed themselves. Hormoslyr was blue as ink. If you cut a potato dumpling to fry, then it was blue. The flowers died at my mother’s home when I went there. […] I guess the fumes were in my clothes.

There seems to have been no clearly articulated opposition to the use of herbicides in forestry at this time, but the speech by the chief forester Fredrik Ebeling at Naturskyddsföreningen’s national convention in 1955 indicates that there must at least have been some discussion of this matter. However, his speech had a polite educational tone, seemingly directed towards people with limited knowledge of the subject of ‘modern’ forestry and does not indicate the presence of any confrontation at that time.

### Broad-scale use of herbicides in Swedish forests in the 1960s

During the second phase, which began in the latter part of the 1950s and ended in the late 1960s, many forest companies began to use herbicides routinely, mainly via cost-effective aerial spraying with airplanes or helicopters. The practice was developed in close collaboration between the forest companies (private and state-owned), chemical producers (e.g., Gullviks AB) and logistics companies specializing in services such as provision of transport and aircraft (e.g., Basbolaget). The correspondence between these actors shows a collegial atmosphere, with people using phrases such as “Honorable brother” and “With the very best wishes from your friend” in otherwise formal business communications. During this period research on herbicides and herbicide use was initiated at the Royal Forestry College (Sw. Kungl Skogshögskolan) and the National Forest Research Institute (Sw. Statens Skogsförsöksanstalt) (Bärring 1965). The research primarily focused on practical aspects and supported use of herbicides in forestry. However, towards the end of this period discussion of human health issues began to emerge, triggered by use of the same herbicide by the USA (called Agent Orange there) in the Vietnam war (Lisberg-Jensen 2006). Retired forester Bertil Stenlund recalled this discussion and his feelings at the time well:

This chemical [hormoslyr—produced by the company BT Kemi] contains two phenoxy acids and traces of dioxin. And then he sits there as a boss [CEO of BT Kemi] and has all these chemists on his staff and not knowing that this substance is dangerous for people’s health… They should have put those guys in jail.

Retired forester Bill Wahlgren also remembers similar feelings when understanding what they were handling:

…and then we talked about it, this substance can for the devil penetrate us just as well as it can penetrate the bark of this alder tree

Another important aspect is that forest workers later in life really became worried about what they had been handling during the years when they worked with the herbicides. Retired forest worker Rolf Konradsson thought a lot about the possible effects:

So I worked with hormoslyr five or six summers…I have really thought about those who were affected [by cancer]…we never washed our hands or anything when we had lunch…

### War in the forest: Protests and conflicts over herbicide use

The third period, beginning in the late 1960s and ending in 1984, was a time of escalating conflict between the forest companies and its allies on one side and a new-born coalition of local activists and environmental organizations on the other. The forest companies had invested a lot of prestige in this. Retired forester Sten-Olov Pettersson recalls how the spraying had temporarily been banned in 1977 and that his company (Domänverket) fought to make it legal again and won.

We were put under severe pressure from the management to find areas to spray then. Our boss said: Now that we have fought so hard for the use of herbcides, then I want you to find areas we can spray.

Initially the forest companies tried to educate and reason with their opponents at meetings and in the media. They argued that: use of herbicides was essential, studies had shown that the herbicides used were harmless to people and the environment, and as professional foresters they had the knowledge to use herbicides safely. Several methods were tried by the foresters to convince people that the used herbicides were harmless. One of the more spectacular ones was to drink the herbicide (hormoslyr, the mix of 2,4-D and 2,4,5-T) in live television and offer reporters to drink it as well (Lisberg Jensen 2006).

It soon became clear that these approaches were not working, but rather provoked more anger and protests. Simonsson et al. (2015) showed that the strength of the emerging environmental movement at this time came as a surprise to many leading foresters and they had to face the fact that non-professionals would not accept their knowledge and arguments. The protests had several driving forces. Göta Andersson, an interviewed villager from Aapua (in Norrbotten), described the motives for the protests in her village as follows:

There were two reasons, first it was about the poison. Most people were against that. But another was that women cleared birches from the forest plantations manually, so they would have lost their jobs. Then there were the berries. People picked them and made money. We sold them and the prices were good.

Nea Matsson, also from Aapua, continued the conversation as follows:

…Especially unemployed women. There were also young people. Our kids picked berries so they could buy their first car. […] It was our berry-picking grounds they were planning to spray.

Magnus Sjögren, engaged in the protest at Sarkavaara near the town of Jokkmokk added an important driving force:

It was the very cynical attitude of the forest company [in this case SCA]

**Fig. 4:**
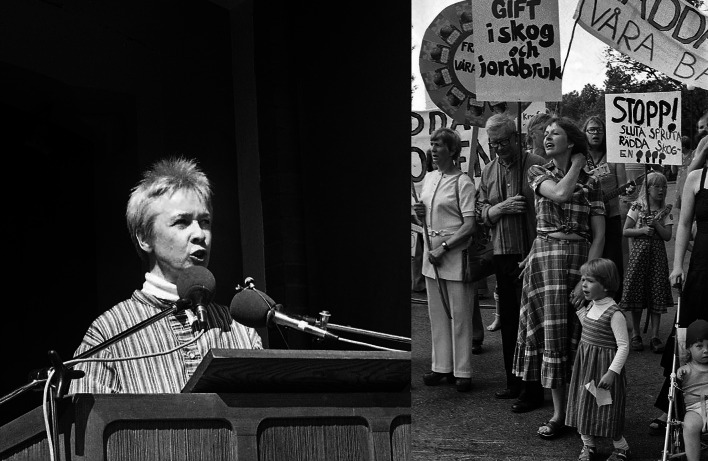
Photographs of writer Sara Lidman giving a speech (left) and demonstration in Torsby, Värmland in 1977 (right) against herbicide spraying in nearby forests owned by the church and commercial forest companies. In her speech Sara Lidman talked about herbicide spraying in the Swedish forests and the herbicide use by the US in the Vietnam war and the need for keeping forestry jobs for women (photos from Värmlandsarkiv, Karlstad)

Clearly, the local protests by people who were not organized or part of national environmental organizations, but rather people with many different motivations, galvanized the campaign to stop herbicide spraying in the forests. Since protests happened simultaneously in many places in northern Sweden the issue soon rose to national prominence. The local involvement at the very sites, which were to be sprayed, was very difficult to counter for the forest companies, both at the local level and at the national level. News on television and photos in newspapers showing “ordinary” people; elderly people, families, kids, forest workers, fighting against large forest corporations on lands next to their villages had a very strong effect on the public opinion and on the politicians. The writer Sara Lidman became a very prominent person and a speaker in the local protests (Fig. 4). She was originally from a small village in northernmost Sweden where Domänverket did extensive herbicide spraying in the 1960s. She was also engaged in the protests against the Vietnam War during the 1960s and 1970s and brought together the concern over the herbicide use by the US. Military in Vietnam with the use of the same herbicide in northern Sweden.

Following confrontation and discussion with Domänverket’s foresters, people in Aapua set up tent camps in the forest and took turns guarding the camp to obstruct the spraying. They also blocked the transport of herbicides and fuel to aircraft.

Evald Matsson from Aapua remembered: “We decided to guard the forest. We set up tents and stayed there all the time.”

Göta Andersson: “…you couldn’t spray areas where there were people. So, we stayed there and made camp fires and picked berries all the time. We took turns. We stayed there for a month, until the end of August when they [Domänverket] gave up and left.”

**Fig. 5:**
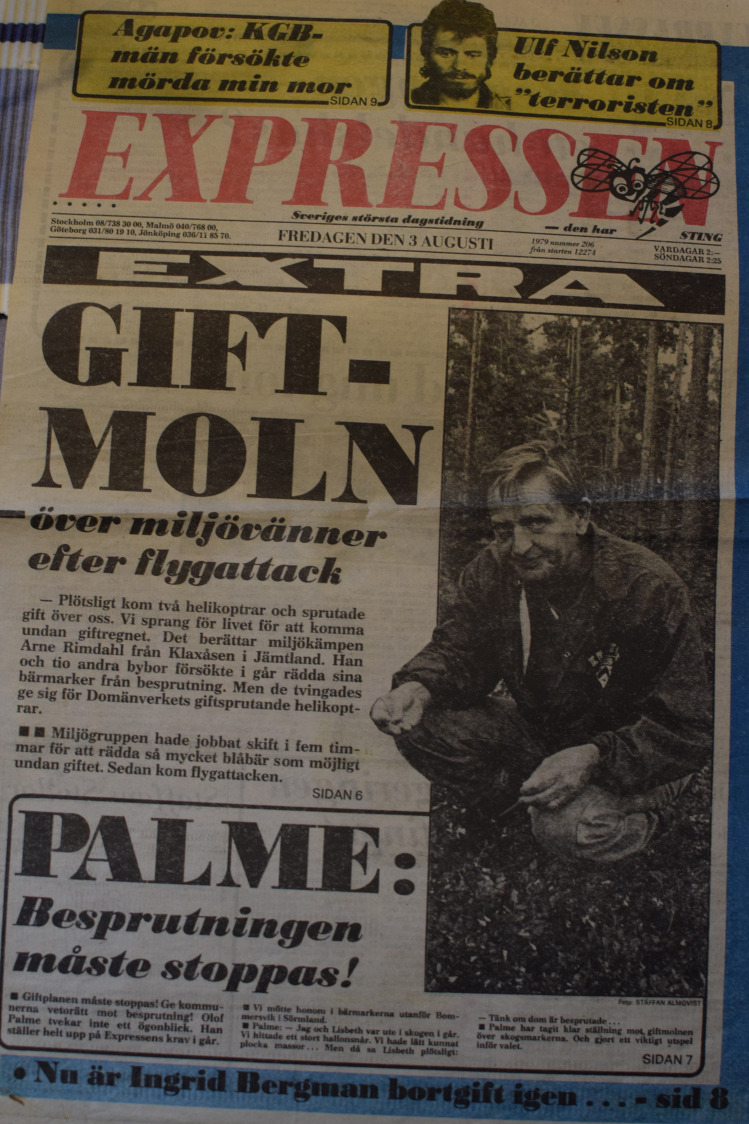
Front page of national tabloid newspaper Expressen featuring prime minister Olof Palme, August 3 1979. Olof Palme states: “The herbicide use must be stopped” (Private archive, Nea and Evald Mattson, Aapua)

Evald Matsson recalled the end of the protests and what happened when they reached politicians:

Prime Minister Olof Palme was out picking berries in the forest and said it might be dangerous to eat these berries. And then it [the spraying with herbicides] ended. (Fig. 5)

Enander (2002, 2007) points to the importance of several leading researchers and their influence on the general debate on chemicals used in the environment as a critical factor. However, our interpretation is that the local protests at many places of planned sprayings, the determination to stop spraying by occupying airfields, blocking transport of fuel and herbicides and self-sacrificing civil obedience were the main driving force and which gave the protests very strong momentum. Strongly increasing media coverage of local protests towards of the 1970s encouraged activists in new places and led to a snowball effect despite the fact that there was no nationally organized movement.

The issues then attracted the interest of medical researchers, who reviewed international studies on phenoxy acids’ potentially harmful effects and initiated studies in Sweden, thereby initiating discussions on the safety of workers who used them (Lisberg-Jensen 2006). Some local activist groups invited these researchers in order to understand the issue better and get arguments in the discussion with the forest companies. Activist Isa Sundberg, then living in Jokkmokk recalls:

We invited people to come and teach us about the poison. We invited for example Dr. Christopher Rappe [a medical researcher] to learn more about this, so we could argue with the forest company

Finally national nature conservation NGO’s became increasingly engaged and brought the protests from the rural areas into major cities and a national discussion emerged. Organizations such as “Fältbiologerna” (a Swedish youth organization of field biologists) and “Naturskyddsföreningen” picked up, reinforced the protests and brought them to new arenas such as major cities. While it all clearly started with local engagement, all of these processes eventually set in motion political processes that the forest companies could not stop, and the use of phenoxy acid herbicides in Swedish forestry was finally prohibited in 1984.

A broader conclusion of this part of our study is that foresters in Sweden could, and did, apply new and potentially harmful methods on a very broad scale with little internal and external opposition during many years. A network of male foresters in senior positions in forest companies, state agencies and research institutions were educated together in the Swedish School of Forestry. A leading, charismatic and very influential forester during this period was Fredrik Ebeling. His motivation to promote large scale use of phenoxy acids in forestry was the idea to transform the old forest in northern Sweden into rejuvenated high productive forest consisting only of trees suitable for the pulpmills and sawmills (Ebeling 1959). He was one of the first to experiment with phenoxy acids, he had a leading position (chief forester) at Domänverket in the 1950s and 1960s, he made numerous public talks where he argued for modern intensive forestry—also “educational” talks directed toward nature conservation organisations—and he finally took the position as CEO at the National Board of Forestry.

Even in the late 1970s there was rather limited scientific discussion regarding use of phenoxy acids in Sweden, especially regarding ecological effects on the forest ecosystems, and few researchers voiced concerns. Those who did had a rather mild tone (cf. Ingelög 1978) and suggested minor limitations in herbicides’ use rather than prohibition. The almost complete lack of internal criticism within the broader forestry community (among both active forest managers and forestry researchers) recorded in documents from this period is both frightening and fascinating.

However, there were a few exceptions. One of the few outspoken opponents of herbicides in the 1960s within the forestry sector was the well-known forester Joel Wretlind, who stated that “Those who set poison to kill them [the birches], yes, well, they should take it themselves” (Cogos et al. 2020). As already mentioned, in the Malå forest district where he was chief, herbicides were hardly used at all during this period.

Another exception during the end of this period was a change in policy by the large commercial forest company Modo AB, which used phenoxy acid herbicides on a moderately large scale during the 1950s and 1960s (Andrén 1992), but decided to end this use completely in 1970. This created tension with other forest companies. They argued that Modo was undermining the essential use of herbicides, and Modo was questioned and openly criticized (S10:5, Andrén 1992). Foresters from Modo AB, in contrast, argued that the use of herbicides was also problematic from a production perspective as it could have detrimental effects on mycorrhizae and trees’ growth. In 1992 the former chief forester Thorsten Andrén concluded that the decision to stop using herbicides in 1970 was “…very far-sighted and, a decision which should have been taken much earlier” (Andrén 1992).

It is striking to listen to one of those active during this time and who now look at the situation in a completely different light. Retired forester Bertil Stenlund summarized his thoughts on this period as follows:

If you look in hindsight at the forestry sector [in Sweden] it’s all very surprising. We were like a sheltered workshop (Sw. skyddad verkstad). During all my years of working there I don’t remember anyone questioning what we were doing. And there really was reason to question it. Here in Lycksele, although this debate about herbicides had started, I never once met forestry workers or anyone else who questioned what we were doing.

Magnus Sjögren, one the activists at Sarkavaara, but who had worked in forestry as a young man, recalls his feelings of using herbicides:

It was this forests which was to be eliminated. In hindsight I am angry that I was so young and didn’t have a network then…. There were large aspen trees (Populus tremuloides) as large as 80 cm in diameter, which we were to kill. I thought no! Damn it! So then I made the notches with the axe, the others will see me since we work together, so I faked it. I made the little notches but I did not pour in the herbicide. So they could live on…].

### The extent of herbicide spraying 1948–1984 and the ecological consequences

The extent of herbicide use in the Swedish forest is impossible to quantify precisely due to the lack of complete historical records and large numbers of actors involved. However, ca. 10% of the three studied forest companies’ forest land was sprayed during the considered period. Moreover, calculations based on the data we compiled from historical records, and contrasted to the national statistics available for 1968–1972, indicate that between 0.7, and 1.1 million ha was sprayed during the period. As several large forest companies used herbicides during the entire period, the total sprayed area was probably closer to the upper end of this range. Thus, despite the lack of complete data, deciduous trees were clearly eradicated from a large area in northern Sweden, with associated impairment of important ecological functions.

Herbicides affect forest biodiversity by causing short-term declines in plant species diversity, changes in vegetative structure, and potentially shifts in plant successional trajectories (Guynn et al. 2004). Moreover, deciduous trees are considered keystone species that carry much of the biological diversity in the conifer-dominated ecosystems of northern Sweden (Hämäläinen et al. 2020; Kivinen et al 2020), so mixed species stands provide more ecosystem services than homogenous coniferous stands (Gamfeldt et al. 2013; Jonsson et al. 2019). Thus, widespread herbicide use to reduce deciduous tree components may have short-term benefits, but result in long-term losses of productivity and ecosystem services, together with increases in vulnerability of the cultivated stands. It is very difficult to fully evaluate the large-scale experiment with herbicides in Sweden, but clearly it had a massive impact on the forest ecosystems. The loss of deciduous trees and ground vegetation across landscapes resulted in biodiversity losses, together with changes in nutrient cycling and successional pathways. However, these changes have received too little attention, and there are urgent needs for more research to understand the local long-term effects of herbicides’ extensive use in Swedish forestry. Retired forester Bertil Stenlund recalled:

I remember being horrified that the rowans [Sorbus aecuparia] disappeared. In those days there where big rowan trees everywhere in the forest. It was like the forest lit up when you came and saw those beautiful trees and their red berries.

And the goat willow trees [Salix caprea] went to hell. The forest became impoverished. I can’t say that I could express it at the time but my feeling was that something awful has happened here.

It would be interesting to compare the extent of phenoxy acid herbicide spraying in Sweden and other countries, but we have not found sufficient records for such comparison. In West Germany, Austria and the DDR, France, Great Britain, Finland and Norway the same phenoxy acids (2,4-D and 2,4,5-T) were used under different product names (Tormona, Selest and Hormest) during approximately the same period (Arbonnier 1957; Donabauer 1963; Willoughby et al. 2009). A superficial survey of forestry journals in these countries indicate that although phenoxy acids have been used in forestry, the extent has been rather limited and not used on a broad landscape scale as they were in Sweden. However, in France, Great Britain and Romania use of phenoxy acids were still allowed in forestry until 2009 (Willoughby et al. 2009), although there are detailed recommendations to minimize it, in Great Britain for example (Willoughby et al. 2004). In Canada broad-scale use of phenoxy acids (2,4-D and 2,4,5-T) continued up until the end of the 1980s, but were contested in courts in many cases (Dunster 1987), and the discussion is still ongoing regarding the use of herbicides in forestry (Freedman et al. 1991; Freedman et al 1993; Charbonneau and Simpson 2011). From the late 1970s, when use of most chlorophoxyacetic acids became restricted, alternative herbicides have been introduced in forestry in many other countries, including glyphosate-based compounds that are now the most commonly used herbicides worldwide (Baylis 2000). In Finland, Norway and most other European countries use of these herbicides on forest land also decreased substantially from the 1980s (Willoughby et al. 2009).

## Conclusion

The study that we present here is broad narrative of a very dramatic period in Swedish forest history. We believe that different methods and sources should be used to convey such a story, which includes forest ecosystems, forest management actions and peoples relation to nature from different viewpoints. Only then is it possible to understand the complex relationship between people and nature.

We conclude that this long episode of Swedish forestry, when deciduous trees were killed on a regional scale has had long-lasting but today largely unknown ecological effects, requires further investigations and discussions. Surprisingly, there has been an almost complete silence within the forestry profession on this topic after the ban on phenoxy acid herbicides in 1984. New research could be directed towards more detailed investigations of the regions where herbicides were most intensively used, then correlate them to National Forest Inventory data (available from 1923 and onwards) and/or current forestry maps. This would allow for a better understanding of the long-term ecological consequences of herbicide use. Also, other tools such as pollen-analysis could be used to investigate the long-term trends (on a centennial scale) of the deciduous component in the conifer dominated forests in north Europe, and how this component was affected regionally during the herbicide period. Such studies would be very useful to establish ecologically sound targets for forestry in the future.

Finally, aside from the obvious conclusion of our study that new and drastic forestry methods should never be applied on a broad scale without thorough analysis of the long-term effect, it is also clear that a too homogenous cadre of like-minded professionals working across commercial companies, state agencies and even universities is dangerous. This might still be the case in Swedish forestry (Andersson and Westholm 2019).

## Supplementary Information

Below is the link to the electronic supplementary material.

Supplementary file1 (PDF 7081 kb)
