# Supplementary material for: The war on deciduous forest: Large-scale herbicide treatment in the Swedish boreal forest 1948 to 1984
Source: Ambio. 2021 Nov 16;51(5):1352–66. doi: 10.1007/s13280-021-01660-5 (PMC8931146; doi:10.1007/s13280-021-01660-5)
Supplement: Supplementary file 1 — Supplementary file1 (PDF 7081 kb) [file 13280_2021_1660_MOESM1_ESM.pdf]

Ambio

Electronic Supplementary Information
This supplementary information has not been peer-reviewed.

Title: **The war on deciduous forest – large-scale herbicide treatment in the Swedish boreal forest 1948 -1984.**

Authors: Lars Östlund, Sandra Laestander, Gerd Aurell, Greger Hörnberg

S1: *Abridged historical background on the use of phenoxy acids in Swedish forestry*

In Swedish forestry the first experiments on phenoxy acid herbicides (chemical compounds acting as growth hormones) were carried out in 1947 (Häggström 1956). The development of these herbicides came from experiments done in Great Britain and the United States during the Second World War and with a purpose to destroy crops (primarily potato-fields) in Germany. The Forest Research Institute of Sweden (Sw. Statens Skogsforskningsinstitut) also showed an early interest in this new area of research. Between the years 1948-1952 researchers Erik Rennerfelt and Pär Fransson published a series of articles on the issue (Bärring 1965). In 1952, The Forest Research Institute accounted for their results from several field studies on herbicide treatments with phenoxy acids to control birch. The field studies were initiated in 1948 within the local districts (Sw. revir) of Domänverket at Pärlälven, Storbacken and Sikå in the Nedre Norrbotten region (Sw. överjägmästardistrikt) (Fransson 1952). The chief forester Fredrik Ebeling at the National Forest (Domänverket AB) was involved in the early development of techniques for manual spraying of herbicides and tools for notching (a method where herbicides are applied to cuts made to the trunk). He was leading actor when it came to converting the old logged-over forest in northern Sweden to intensively managed production forests (Ebeling 1959). The Forest Research Institute recognized several advantages in using phenoxy acids to control birch and thus in order to favour the more economically valuable conifers Scots pine and Norway spruce. The main advantages were that phenoxy acids were selective herbicides, they were not toxic to grass or cereals, they were supposedly be harmless to people and animals and they were more cost-effective compared to the traditional manual clearing of trees. Based on the results from the field studies the Forest Research Institute gave specific directions on how to control birch with herbicides (Fransson 1952). The directions informed about appropriate preparations and mixtures of herbicides, about favourable time for application and weather conditions. Recommended treatments were spraying of birch less than 3-4 meters of height, spraying of shoots from stumps, spraying of brushwood and small trees. For mature and old deciduous trees, the herbicide was poured into pockets made into the trunk with a special axe with a concave head holding a sufficient amount of the herbicide. Furthermore, the directions included which measures that had to be taken when deciduous trees were sprayed with herbicides in sites with already present regeneration of Scots pine (*Pinus sylvestris* L.) and Norway spruce (*Picea abies* (L.) Karst.).

In 1955 Ulf Bärring at the Royal Forestry College (Sw. Skogshögskolan) took command of the experimental work with herbicides against deciduous vegetation. In 1965 Bärring published a report about treatment of deciduous trees with herbicides based upon results from the experimental work (Bärring 1965). The report gave a description of the different application methods that had been developed; which were foliage spraying, basal bark spraying, stump spraying and notching. Foliage spraying could either be done from the ground or aerially, and it was the prevalent application method to control deciduous vegetation since it was the most cost-effective method. In contrast to notching, where individual trees were treated with undiluted herbicide, foliage spraying were distributed over large areas and water or diesel were used as carrier and to dilute the herbicide (Anon. 1974).

Foliage spraying and notching were carried out from two to three weeks after the leafs had sprouted on the trees and until the end of the summer. In southern Sweden the herbicide spraying could thus start about the 1st of June and continue to about the 15th of August and in northern Sweden sprayings could be performed from about the 15th of June until the 1th of August (Anon. 1974). In young stands with presence of conifers it was recommended that herbicide sprayings were carried out after breaking of the buds, in order to avoid damages to the apical shoots on the conifer trees (Anon. 1974).

The most used phenoxy acids in Swedish forestry were 2,4-D, dichlorophenoxyacetic acid, 2,4,5-T, trichlorophenoxyacetic acid and MCPA, 2-methyl-4-chlorophenoxyacetic acid (Anon. 1974, Lisberg Jensen 2006). In the production of the phenoxy acid 2,4,5-T some contaminants were formed, of which the dioxin 2,3,7,8-TCDD was the most toxic substance (Lilienfeld & Gallo 1989). The susceptibility to phenoxy herbicides varied among different species. 2,4-D was considered to be the most appropriate herbicide against sensitive species such as alder (*Alnus* spp. Mill), birch and goat willow (*Salix caprea* L.) whereas 2,4,5-T was more effective against resistant species such as oak (*Quercus* spp. L.) and other nemoral decidous species (Anon. 1974). The products that were used on the market often consisted of a 2:1 mixture of 2,4-D and 2,4,5-T. One of the most widely known preparations; *Hormoslyr 64*, consisted of a mixture of 2,4-D and 2,4,5-T in ester form.

At the beginning, herbicide sprayings were primarily carried out from the ground, but already in 1951, the first aerial sprayings with herbicides were tried by the commercial forest company Kramfors AB within a small experiment area covering about 70 ha (Lundberg 1952, Bärring 1965). The herbicide sprayings were accomplished by Basbolaget, a company established in Stockholm specialized in controlling different kinds of “pests” in forests and on agricultural lands. Basbolaget had one division for plant protection, one division for timber protection and one division for forest protection. Although Basbolaget had a very dominant position, they considered that they kept their objectivity towards the market by letting the customers decide which preparation to use in the herbicide treatments.

Before an aerial spraying could take thorough preparations had to be done. In advance, Basbolaget sent out instructions about how the preparation work should be done to the responsible district forester. Until 1955 the following points were included in the preparation work; 1) marking out lines with poles at intervals of 30 meters, in order to show the flight route, 2) clearing and marking out an approximately 2-meter broad lane so the signallers could move between the poles (the signallers used balloons to help the pilot orient along the flight route), 3) arrange landing grounds for the helicopters on level and solid ground with access to clean water within 100 meters from the landing ground, 4) arrange transportation roads, 5) prepare maps of the area on the scale of 1:20 000, 6) make requirements on preparations and dosage per hectare, 7) determine the exact area that was planned to be sprayed with herbicides, 8) arrange with assistance staff, to each helicopter 6 signallers and 2 persons for filling up water were required and, 9) arrange transportation of preparations, oil, petrol and hydrogen gas to the spraying area.

In typical contracts between Basbolaget and Domänverket it was specified that herbicide spraying would be carried out in the period of 20th June to 1st of August during favourable weather conditions, which were light breeze, light thermals and dry weather (Laestander 2015). From 1956 aerial sprayings generally were performed by “free flight”, which meant that the pilot made the orientation by means of natural landmarks. If there were not enough natural landmarks within the spraying area fixed signals in form of flags were prepared in advance.

During the first years the aerial sprayings showed varying results and the Forest Research Institute initiated studies of areas that had been aerially sprayed with herbicides in order to establish which factors that had the greatest influence on the spraying results (Bärring 1965). The results from the studies showed that proper dispersion of the spraying liquid was one of the most crucial factors for the result of the herbicide treatment. The dispersion of the spraying liquid was affected by wind and thermal conditions, where light breeze and light thermals close to the ground were the most favourable conditions. Bärring’s (1965) recommendations for successful herbicide spraying were that the treatment area should have similar topography, that the conifers should be felled before herbicide spraying in mature stands with a high proportion of deciduous trees and that the aerial spraying should be performed at the lowest height over ground as possible. A low flight height could be facilitated by cutting overstory trees before herbicide spraying.

In the middle of the 1950’s, the forestry committee of Norrland for phenoxy herbicides (Sw. Norrlands skogsvårdsförbunds kommitté för hormonderivatfrågor) and Domänverket made follow-ups on aerial sprayings to determine which factors had the greatest influence on the spraying results (Kungliga Domänstyrelsen 1955). Their conclusion was that the temperature after herbicide spraying was of importance and that high temperatures improved the effect of the herbicides. One factor that was considered to negatively affect the result of the herbicide spraying was precipitation in connection with the spraying. Domänverket assessed that sprayings that were performed early in the summer had a better effect than sprayings that were carried out later with the same herbicide dosage. They also recommended that the width of the flight routes should be reduced to 15 meters and they pointed out that it was important to follow the terrain carefully during flight (Kungliga Domänstyrelsen 1955). Domänverket compiled directions for correct herbicide dosage at aerial spraying based on which point of time in the summer the spraying would be done and at which altitude the treatment area was situated (Kungliga Domänstyrelsen 1955).

In a more recent report about the distribution of herbicides (Anon. 1974), it was stated that a light and steady breeze between 1-4 meters per second was better than calm weather at the time of the herbicide spraying. Calm weather conditions increased the risk that rising air streams spread the preparations outside of the treatment area. In the report it was also pointed out that because of thermals during sunny days, aerial spraying was most frequently done during early morning, in the evening and in the night.

Foliage spraying was the primary method for spreading herbicides and the method used in aerial spraying (Anon. 1974). In 1970, Domänverket estimated the costs for foliage spraying

with different application methods. In northern Sweden the cost for aerial spraying was estimated to be 38 Swedish crowns (SEK) per hectare, tractor spraying was estimated to cost 61 SEK per hectare and manual spraying was estimated to cost between 100 and 400 SEK per hectare (Anon. 1974). It was obvious that aerial spraying was the cheapest method for spreading herbicides and for that reason the proportion of forest land treated from the air increased, compared with the area treated from the ground (Anon. 1974). In 1970, 58 percent of the herbicide treated area in Sweden were sprayed from the air, 21 percent were treated manually, 7 percent were sprayed using tractor and 14 percent of the area were treated by notching (Anon. 1974).

The use of phenoxy acid herbicides in forestry in Sweden did not attract much attention during the 1950s and 1960s. However, in the early 1970s, environmental concern and a public debate emerged (Simonsson et al 2014). The debate had its main roots in the criticism against pesticides and herbicides, but a number of other management practices within forestry were also criticized, including clear-cutting, fertilisation, scarification, the usage of DDT and planting of exotic tree species (Enander 2007b, Simonsson et al. 2014). The criticism against the use of herbicides were principally based on the uncertainties about possible health risks and on suspicions that diseases and deaths among humans and animals were connected with the distribution of herbicides (Enander 2007a). The protests were especially in the beginning happening in rural areas where the spraying with herbicides took place. One event that attracted much attention was a report from the region of Älvsbyn in Norrbotten, that a number of reindeer had passed away after browsing in an area that had been sprayed with phenoxy acids (Bovey & Young 1980). The Swedish National Veterinary Institute (Sw. Statens Veterinärmedicinska anstalt) investigated samples taken from the dead reindeer and their conclusion was that the primary cause of the deaths of the reindeer were starvation (Enander 2007b). Despite this uncertainties about health effects and possible risks with herbicides remained and was an important driving force in the debate.

Because of the uncertainties about possible health risks with herbicides, in 1970, a state agency “Giftnämnden” (the “Poison Agency”) set up a group of experts to analyse the health risks with phenoxy herbicides (Giftnämnden 1991). In February 1971 the group delivered their results in a white-book. The verdict from the expert group was that harmful effects on animals and humans had been proved only at doses much higher than the normal use doses of the different preparations (Anon. 1975). However, the expert group considered that further research was necessary and, for that reason the commission suggested a temporary ban in the use of phenoxy herbicides against deciduous vegetation in forestry. This ban was modified in February 1972 and certain application methods for herbicides were permitted (Enander 2007a, Lisberg Jensen 2006). After intensive protests, the Swedish Parliament at the 26th of April 1972 instituted a law that prohibited distribution of herbicides from the air with an exception of arable land (Anon. 1974). In the forest, herbicide spraying from the ground and notching were still allowed.

In 1974 and 1975 the Swedish Environmental Protection Agency (Sw. Naturvårdsverket) published two white books about the ecological effects of phenoxy herbicides and their effects on placental mammals and humans. The white books showed that vegetation that had

been normally sprayed with phenoxy herbicides did not cause any acute toxic symptoms for wildlife. The effects of phenoxy herbicides on insects, for example bees, were not adequately analysed. Despite all reports of diseases and deaths in connection with herbicide sprayings, no confirmed poisoning had been identified in humans (Enander 2007b). However, in the white book about herbicides and their effects on mammals and humans it was established that further research about phenoxy herbicides and their potentially carcinogenic and genetic effects were necessary (Enander 2007a).

Even though the white books concluded that phenoxy herbicides had not caused any confirmed poisonings in humans, the mistrust of herbicides were additionally strengthened by reports of the effects of the preparations on humans and environment during the Vietnam war (Enander 2007a). During the war in Vietnam the preparation Agent Orange, which was a mixture of equal parts of 2,4-D and 2,4,5-T, was used by the US Army for defoliation for strategic war purposes (Lilienfeld & Gallo 1989). 2,4-D and 2,4,5-T were the same phenoxy acids that constituted the preparation *Hormoslyr 64*, which was used for herbicide spraying in Swedish forestry. The reports from Vietnam described an increased frequency of foetal damages after the dispersion of Agent Orange because of the toxic substance dioxin, which was present in the phenoxy acid 2,4,5-T (Enander 2007a). A commission set up by the US Academy of Science established that the data used for the studies made it impossible to draw any reliable conclusions and that the liaison between Agent Orange and foetal damages could not be proven (Lilienfeld & Gallo 1989). However, there has been several lawsuits where both producers as well as the US government have accepted to pay damage to those affected to Agent Orange. The many turns in this question this did not diminish the concerns among the public, neither in Vietnam, the US or Sweden. If anything, the public discussion and the scientific reports strengthened the protesters in Sweden further. Later on, the long-term effects of for workers exposed to these chemicals have continued to be debated and caused concern and worry. One specific and well known case is the village Jutis, west of Arjeplog in Norrbotten, named “the village of widows” due to the high number of cancer cases among forest workers (https://www.svtplay.se/video/28739618/kanal-3/jutis-ankornas-by).

In 1974 a report about the general use of herbicides in forestry was published and the report recommended that the ban on aerial spreading of herbicides was to be discontinued. Based on the report the Swedish Parliament decided that aerial spraying of herbicides was to be allowed again from the 1st of July 1975. During the summer of 1975, many local protests including various forms of civil disobedience against aerial spraying of herbicides arose which in turn caused intense media debate both in local newspapers and national newspapers as well as in television and radio. After more protests and an increased uncertainty about the phenoxy herbicides and their actual health effects, the phenoxy acid 2,4,5-T, which was a part of *Hormoslyr 64*, was prohibited in 1977 (Laestander 2015). In 1979 a parliamentary inquiry about the use of chemicals within forestry was set up and consequently a temporary ban for 1980 and 1981, on all spreading of herbicides against deciduous vegetation in the forest, was decreed. The parliamentary inquiry led to an act on chemical herbicides for deciduous vegetation that came into force in 1983. However, because of a change of Government in the autumn of 1982, the act had a very short duration. A new act came into force on the 1st

January 1984 and stipulated a general ban on the distribution of chemical preparations over forest land (Enander 2007a). It was possible to apply for exemptions at the Swedish Forest Agency (Sw. skogsvårdsstyrelse), but after the introduction of the act, the extent of herbicide treatments was reduced. No aerial spraying with herbicides has occurred in Sweden after the year of 1986.

Literature ESM

Andrén, T. (1992) Från naturskog till kulturskog – Mo och Domsjö AB:s skogsbruk under ¾ sekel 1900-1979. CEWE-förlaget, Bjästa.

Anon. 1974. Spridning av kemiska medel. Betänkande avgivet av utredningen om spridning av kemiska medel. Sveriges riksdag, Stockholm. SOU 1974:35.

Anon. 1975. *Information om fenoxisyror*. Statens Naturvårdsverk. SNV PM 629. Produktkontrollbyrån, Stockholm.

Anon.1976. Kemisk lövslybekämpning i skogsbruket. Domänverket, Skogsindustriernas Samarbetsutskott, Sveriges Skogsägareföreningars Riksförbund. Stockholm, 8 pp.

Bovey, R.W. & Young, A.L. 1980. The science of 2,4,5-T and associated phenoxy herbicides. John Wiley & Sons, New York.

Bärring, U. 1965. Behandling av lövträdsvegetation med herbicider. Studia Forestalia Suecia Nr 25. Skogshögskolan, Stockholm.

Bärring, U. S. M. (1978). The Use of Phenoxy Herbicides in Swedish Forestry: Amounts, Types, and Modes of Application. Ecological Bulletins, 219-230.

Dunster, J.A. 1987. Chemicals in Canadian Forestry: The Controversy Continues. Ambio 16 (2/3 Forestry):142-148.

Ebeling, F (1959) Skogarnas och deras vård i övre Norrland från och med 1930-talet. In Arpi (ed) Sveriges skogar under 100 år, del II. Ivar Haeggströms boktryckeri AB, Stockholm.

Enander, K.-G. 2002. Framväxten av en skoglig miljöpolitik. In: Skogshistoriska sällskapets årsskrift: 98-119.

Enander, K.-G. 2007a. *Skogsbruk på samhällets villkor: Skogsskötsel och skogspolitik under 150 år*. SLU, Umeå. Rapport 1:2007.

Enander, K.-G. 2007b. *Ekologi, skog och miljö: vetenskap och idéer under 300 år*. SLU, Umeå. Rapport 4:2007.

Fransson, P. (1952) Bekämpning av björk i Norrland medelst hormonderivat. Meddelanden från staten skogsförsöksanstalt. 41 (1): 1-19

Giftnämnden (1991). Fenoxisyror: Granskning av aktuell information. Rapport från en expertgrupp.

Häggström, B. 1956. Om hormonpreparat och deras användning. Sveriges

skogsvårdsförbunds tidskrift, s 240-247.

Ingelög, T. (1978). Effects of the silvicultural use of phenoxy acid herbicides on forest vegetation in Sweden. Ecological Bulletins, 240-254.

Kungliga Domänstyrelsen, 1955. Cirkulärskrivelse nr 11.

Lautehschlager, R.A. & Sullivan, T.P. (2002) Effects of herbicide treatments on biotic components in regenerating northern forests. The Forestry Chronicle 78: 695-731.

Laestander, S. (2015) Den kemiska bekämpningen av skadlig lövskog har öppnat helt nya vyer för skogsbruket – Flygbesprutning med herbicider i Arjeplog 1953-1978. Examensarbete i biologi (2015:7). Fakulteten för skogsvetenskap. Institutionen för skogens ekologi och skötsel. Umeå.

Lilienfeld, D.E. & Gallo, M.A. 1989. 2,4-D, 2,4,5-T and 2,3,7,8-TCDD: An overview. Epidemiologic Reviews 11: 28-58.

Lisberg Jensen, E. 2006. Sätt stopp för sprutet! Från arbetsmiljöproblem till ekologisk risk i 1970-talets debatt om hormoslyr och DDT i skogsbruket. I: Miljöhistoria över gränser; Skrifter med historiska perspektiv, s 197-230. Malmö högskola.

Miller, K.V. & Miller, J.H. (2004) Forestry herbicide influences on biodiversity and wildlife habitat in southern forests. Wildlife Society Bulletin 32:1049-1060.

Östlund, L., Zackrisson, O. & Axelsson, A.-L. 1997. The history and transformation of a Scandinavian boreal forest landscape since the 19th century. Canadian Journal of Forest Research, 27:1198-1206.

Östlund, L. & Zackrisson, O. (2000) The forest history of boreal Sweden: a multidisciplinary approach. I: Agnoletti, M. & Anderson, S. (eds.). Methods and approaches in forest history. 119-128. CABI Publishing, Wallingford; Great Britain.

Simonson, P., Gustafsson, L. & Östlund, L. 2014. Retention forestry in Sweden: driving forces, debate and implementation 1968-2003. Scandinavian Journal of Forest Research 1-20.

S2: Chemical control of deciduous trees [Kemisk lövbekämpning] – movie produced by the National Board of Forestry, Sweden. From the National archive at Härnösand, Kungliga Domänstyrelsen, photos and movies. Available online at: https://svelantbruksuniversitet-my.sharepoint.com/:v:/g/personal/gustaf_egnell_slu_se/Ed1R2M4ylzVEkpLRBoOIuygBTUelhVXf8jd_XcpfrF0Elg?e=vihngQ

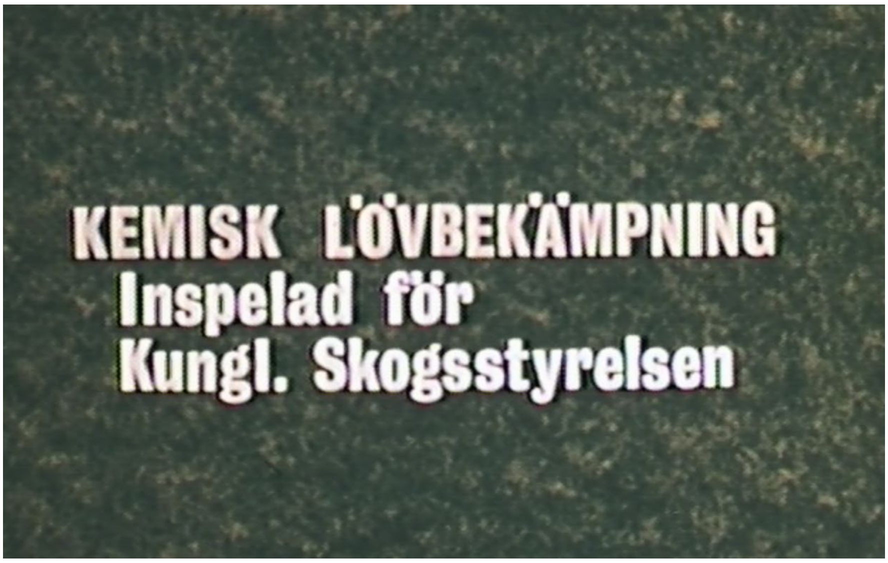

S3: Pamphlet produced by SCA AB to inform the public about phenoxy acids, why they were used in forestry and that there is no reason to be afraid of these chemicals. *"Questions and answers about phenoxy acids. Information from the forestry division at SCA. Phenoxy acids are used on some clear cuts to hamper the growth of deciduous trees, which threaten to choke the valuable pine- and spruce-seedlings"*

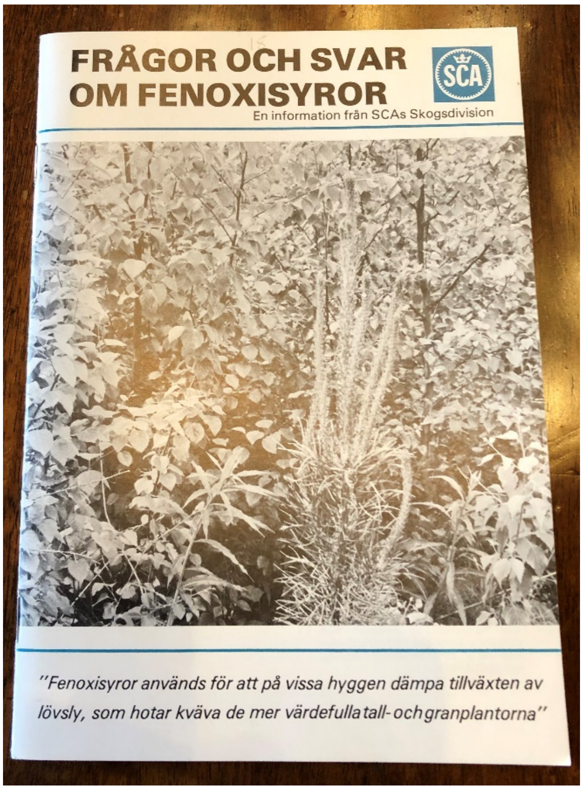

S4: Poster produced by the chemical company Gullviks AB showing available commercial chemicals used in forestry and their use against plants, trees and animals, and also the different application methods of these chemicals. Archived at the Swedish University of Agricultural Sciences, department of Forest Ecology and Management, Umeå.

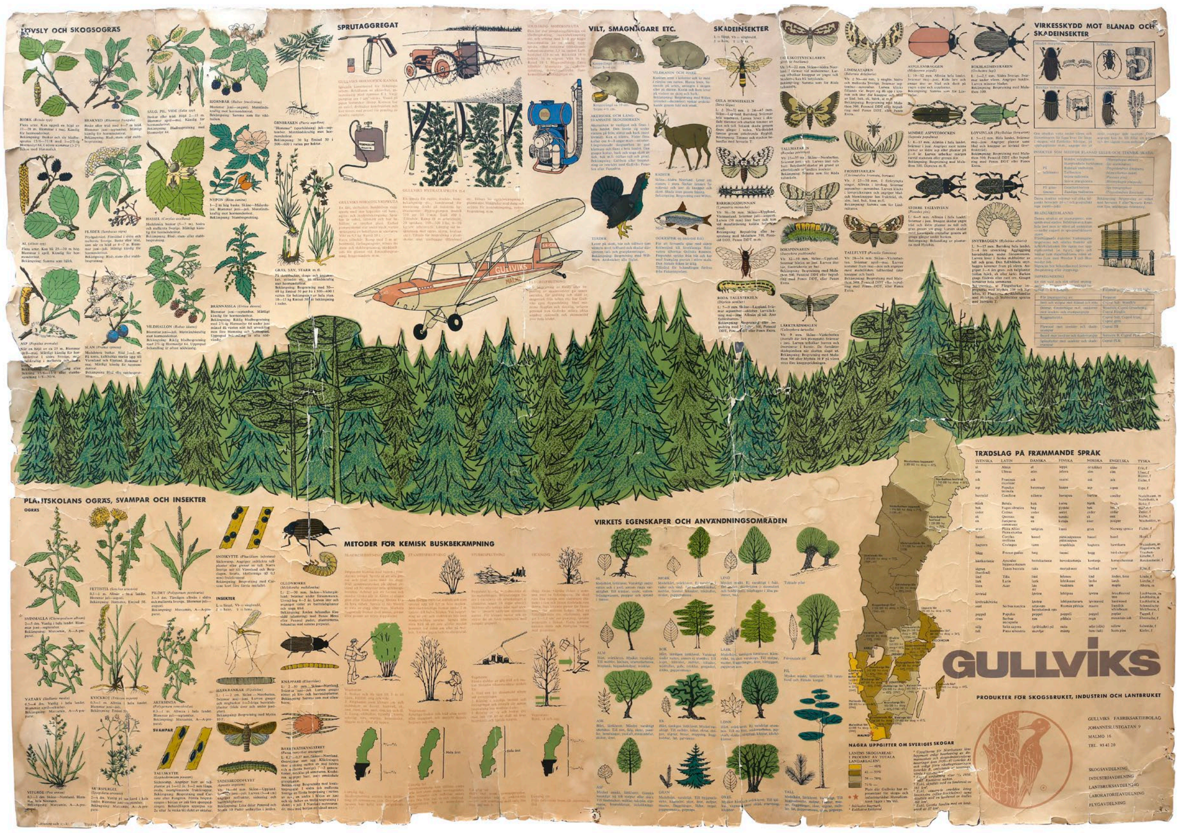

S5a: Map showing forest stand in Arjeplog, near the lake Kronlund (owned by Domänverket) and where larger birches were killed 1960 by notching and injection of herbicide.

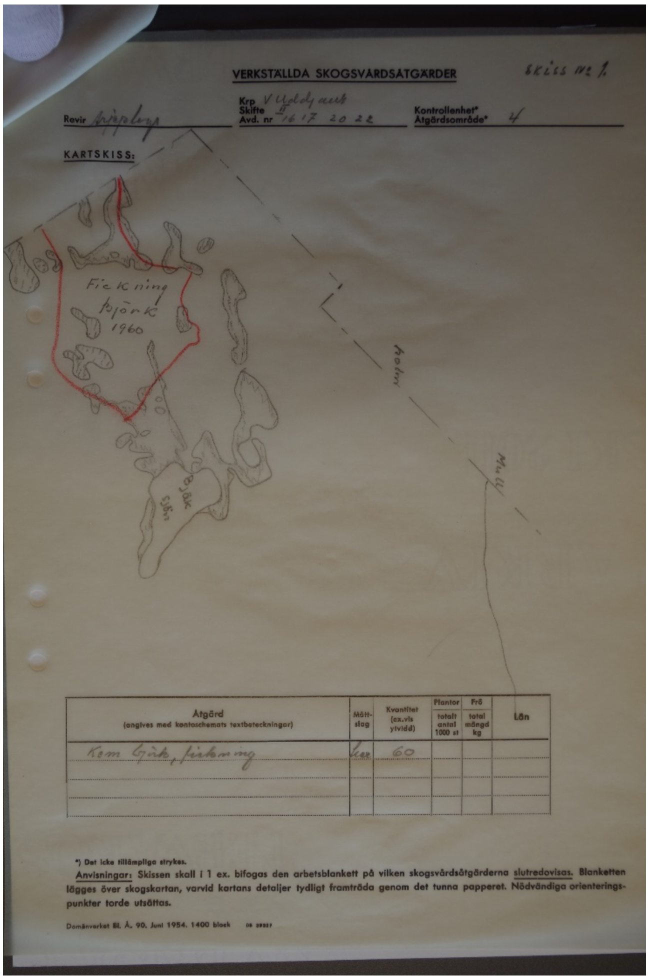

VERKSTÄLLDA SKOGSVÅRDSÅTGÄRDER — Skiss nr 1.

Revir Arjeplog — Krp — Skifte — Avd. nr 1617 2022 — Kontrollenhet* Åtgärdsområde* 4

KARTSKISS:

Åtgärd (angives med kontoschemats textbeteckningar)	Mått-slag	Kvantitet (ex.vis ytvidd)	Plantor totalt antal 1000 st	Frö total mängd kg	Lön
Kem [illegible], fickning	[illegible]	60			

*) Det icke tillämpliga strykes.

Anvisningar: Skissen skall i 1 ex. bifogas den arbetsblankett på vilken skogsvårdsåtgärderna slutredovisas. Blanketten lägges över skogskartan, varvid kartans detaljer tydligt framträda genom det tunna papperet. Nödvändiga orienteringspunkter torde utsättas.

Domänverket Bl. Å. 90. Juni 1954. 1400 block

S5b: The chemical *"Herbexon special"* commonly used to kill larger trees by notching and inserting the undiluted chemical into the trunk of the tree.

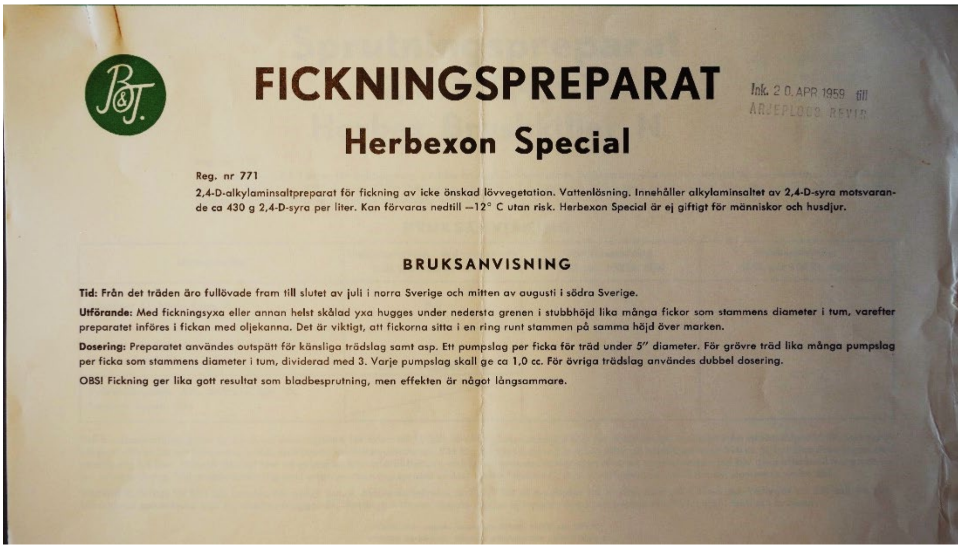

FICKNINGSPREPARAT

Ink. 20 APR 1959 till ARJEPLOGS REVIR

Herbexon Special

Reg. nr 771

2,4-D-alkylaminsaltpreparat för fickning av icke önskad lövvegetation. Vattenlösning. Innehåller alkylaminsaltet av 2,4-D-syra motsvarande ca 430 g 2,4-D-syra per liter. Kan förvaras nedtill —12° C utan risk. Herbexon Special är ej giftigt för människor och husdjur.

BRUKSANVISNING

Tid: Från det träden äro fullövade fram till slutet av juli i norra Sverige och mitten av augusti i södra Sverige.

Utförande: Med fickningsyxa eller annan helst skålad yxa hugges under nedersta grenen i stubbhöjd lika många fickor som stammens diameter i tum, varefter preparatet införes i fickan med oljekanna. Det är viktigt, att fickorna sitta i en ring runt stammen på samma höjd över marken.

Dosering: Preparatet användes outspätt för känsliga trädslag samt asp. Ett pumpslag per ficka för träd under 5" diameter. För grövre träd lika många pumpslag per ficka som stammens diameter i tum, dividerad med 3. Varje pumpslag skall ge ca 1,0 cc. För övriga trädslag användes dubbel dosering.

OBS! Fickning ger lika gott resultat som bladbesprutning, men effekten är något långsammare.

S6: Map showing area which was sprayed with herbicides in 1953 and then again in 1956 within Arjeplog district of Domänverket at Rengård near Torborg. Red lines show the extent of the herbicide treatment and straight lines within the area show flight paths for the airplane.

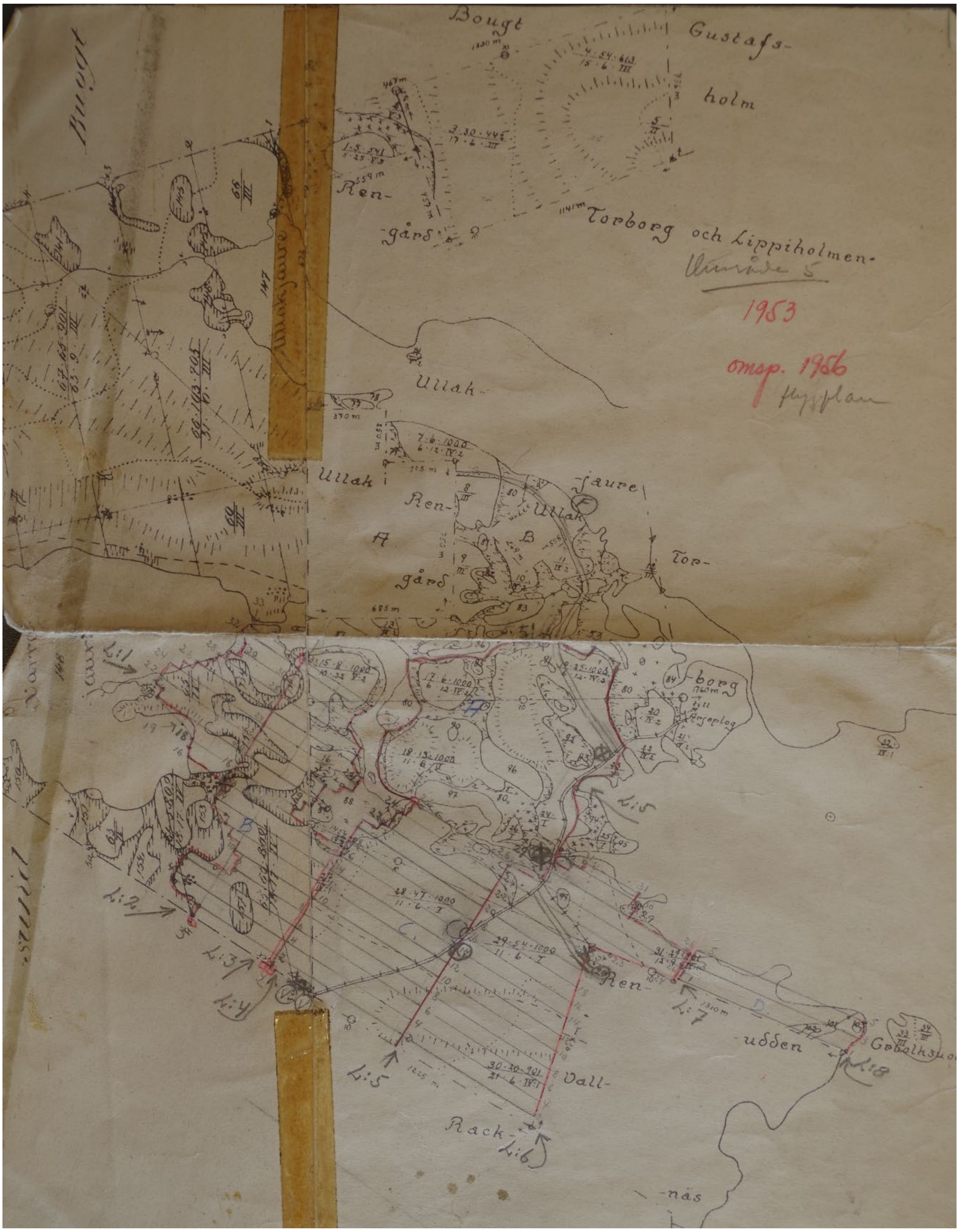

S7a-c: The conflict between the national forest company Domänverket AB and the villagers in Aapua, Norrbotten in August 1979. Photos from the private archive of Nea and Evald Mattson, Aapua.

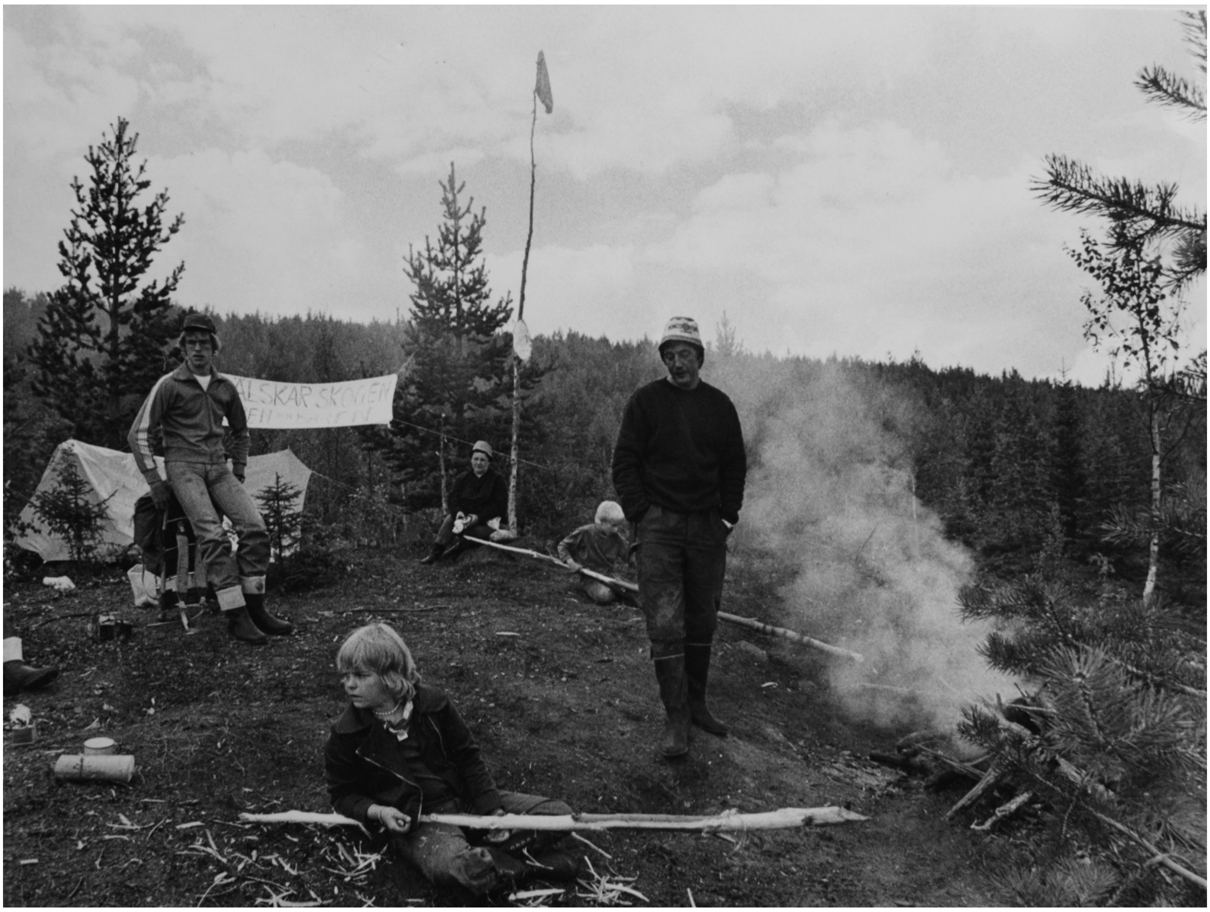

a) The camp outside Aapua set up by villagers to stop the herbicide spraying. The banner in the background states: *"We love the forest, the animals and the berries"*.

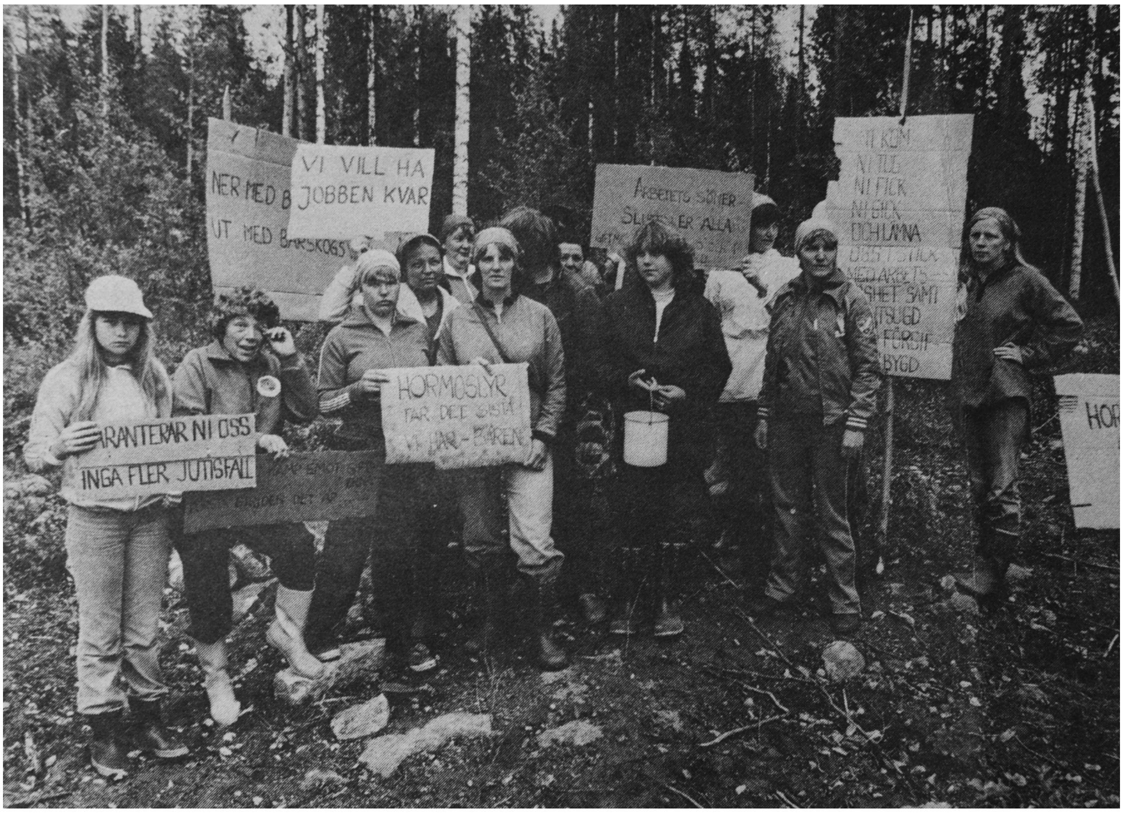

b) Women in Aapua protesting against the herbicide spraying outside the village with the arguments *"Keep our jobs"*, *"Save the berries"*, and *"No guarantees to avoid cancer"*.

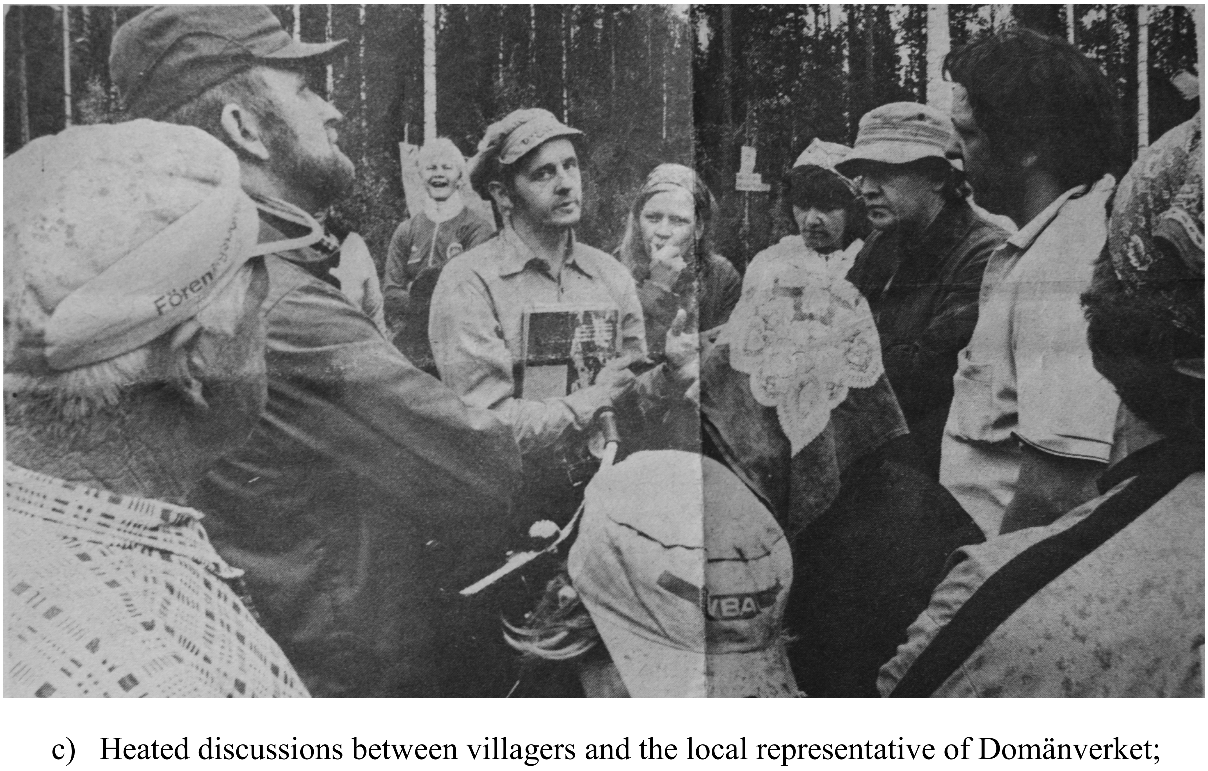

c) Heated discussions between villagers and the local representative of Domänverket; forester Kurt Granberg (in the middle of the picture, carrying a book)

S8: Dead birches after herbicide spraying at Malgomajs revir, Västerbotten in a forest owned by Domänverket AB in 1960. National archive, Härnösand, Kungliga Domänstyrelsen, photos and movies.

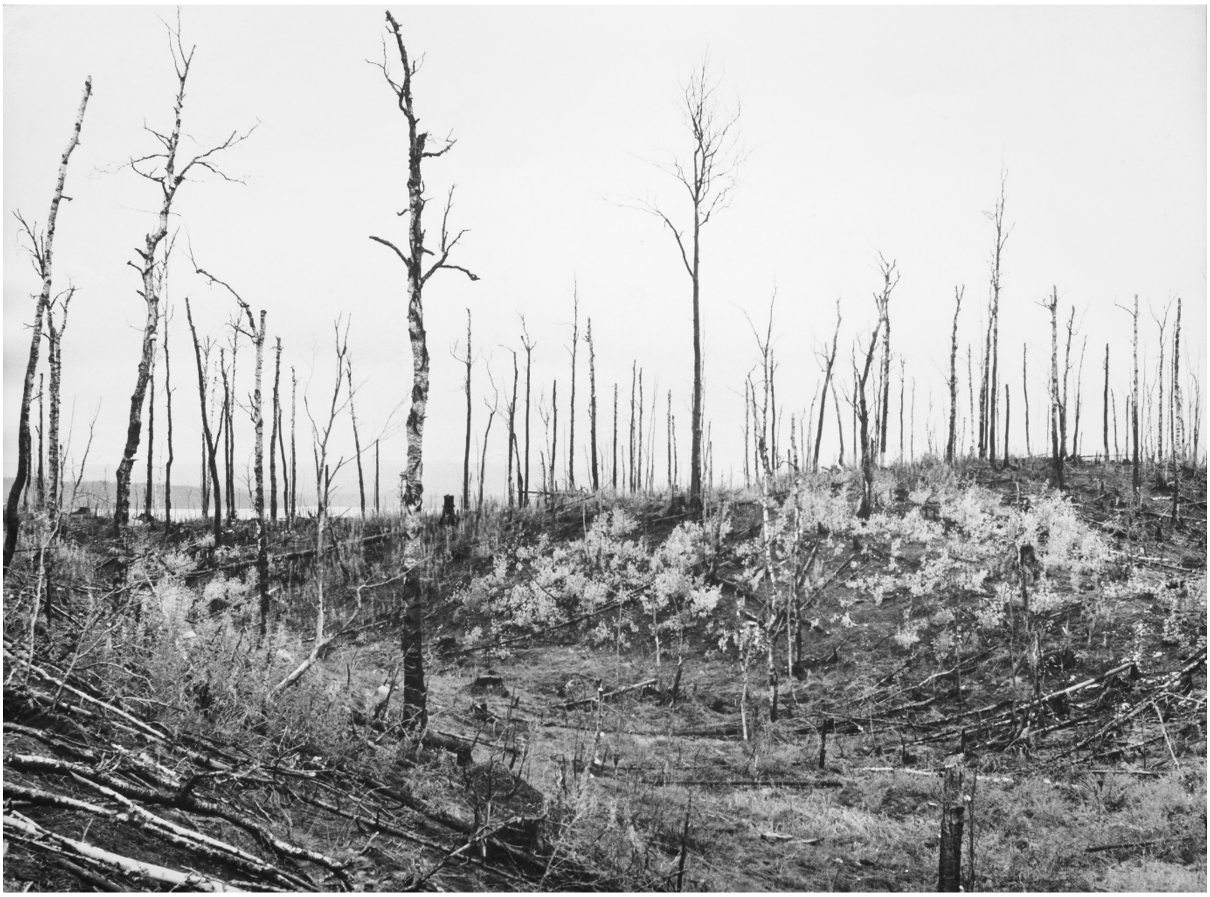

S9: Protests against herbicide spraying in Torsby in 1977. Text on banner: “Save the forest – the environment for our children”. Archived at Värmlandsarkiv, Karlstad.

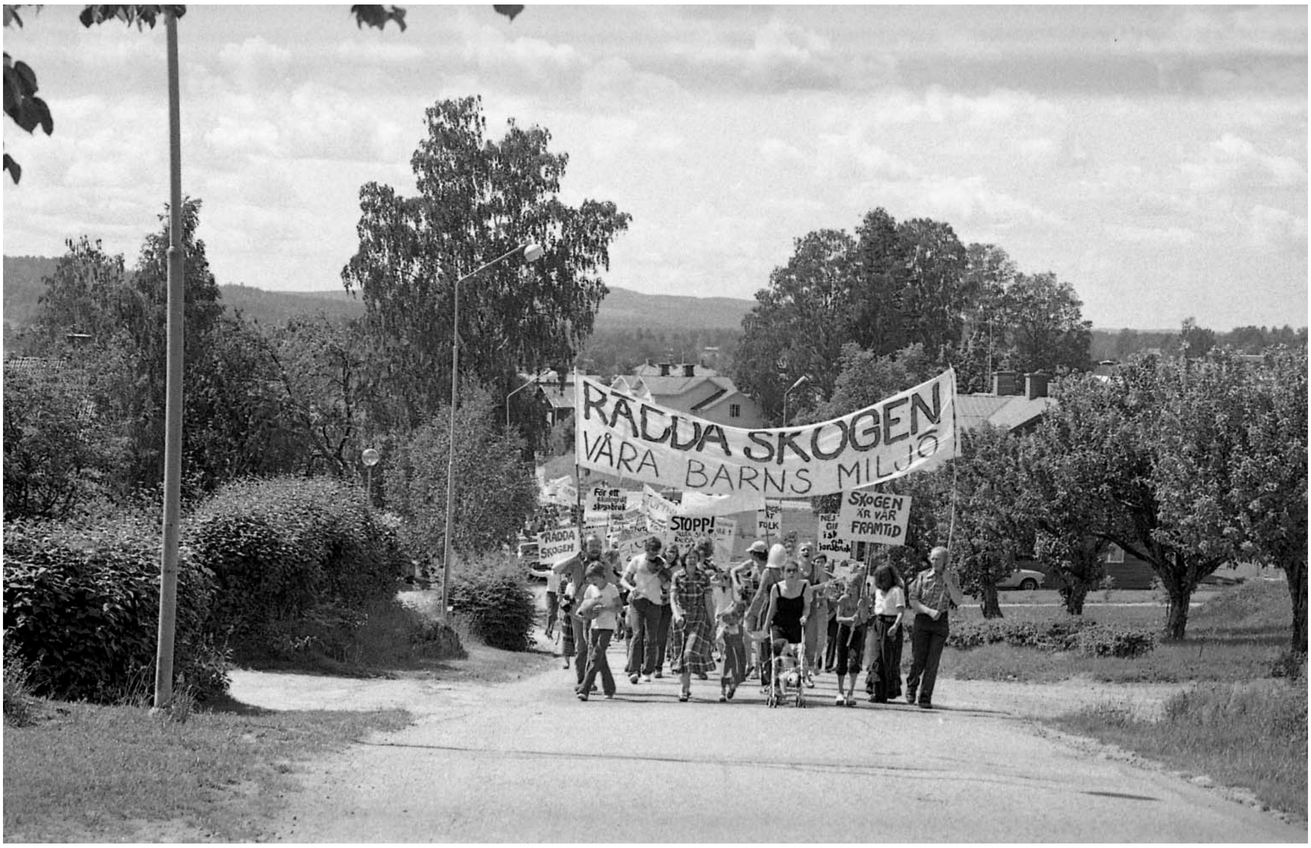

S10: Appendix 1. Historical records used in the study with English translations. Numbers in bold after each archival material is the number used in the article text to identify the records.

Riksarkivet (The Swedish National archive, Härnösand)

- Kungl. Domänstyrelsens arkiv (the Royal National forest archive)
  - Jägmästarnas årsberättelser (Foresters reports) 1948-1984 **(1)**
  - Skogsvårdsstatistik (Forestry statistics) 1967-1978 **(2)**
  - SI Skogsvårdsavdelningen (Forestry department) **(3)**
    - Byråchef Hans Olssons efterlämnade handlingar; pesticider 1949-1974 (Chief Hans Olssons documents: pesticides) **(4)**

  - Domänverket, distriktsförvaltningen (The National forest, district management)
    - Umeå region (Umeå)
      - Bekämpningsmedel, kemiska preparat 1974-78 F XII (Pesticides, chemicals) **(5)**
    - Luleå region (Luleå)
      - Sv 50, 50.10, Skogsvård (Forestry) **(6)**
      - Bekämpningsmedel, Kemiska preparat, 1974-1989 FXII:156 (Pesticides, chemicals) **(7)**
    - Arjeplogs revir (district)
      - Sakordshandlingar, Skogsvård (documents, forestry
        - Björkbekämpning 1953-56 EII20 (eradication of birches **(8)**
        - Björkbekämpning 1957-60 EII21 (eradication of birches **(9)**
        - Skogsvård, åtgärder 1957-1969 EII22-EII29 (forestry actions) **(10)**
      - Ämnesordnade handlingar (documents)
        - Skogsvård 1970-1981 F XII (Forestry) **(11)**
    - Lycksele revir (district)
      - Skogsvård 1949-1978 (Forestry) **(12)**
      - Årsberättelser (Yearly reports) 1958-1970 **(13)**
      - Flygbesprutning 1961-1970 (aerial spraying) **(14)**
      - Kemisk bekämpning 1971-1980 (chemical treatment **(15)**

    - Pajala revir (district)
      - Skogsvård, Bekämpningsmedel 1970-1985 (forestry pesticides) **(16)**
      - Allmänna ärenden 1958-1968 (public matters **(17)**
      - Flygbesprutningar 1960-1969 (aerial spraying) **(18)**
      - Skogsvård FXII (1967-1991) vol 49-51 (Forestry) **(19)**
    - Tärendö revir (disctrict, also partly Pajala och Muonio districts)

Skogsvårdshandlingar 1958-1968 (forestry documents) **(20)**
Skogsvårdsskisser 1960-1970 (forestry sketches) **(21)**

Merlo-arkivet, Sundsvallsbolagen (The Merlo-archive, Sundsvall forest companies)
SCA AB, Skogsavdelningen, årsberättelser 1948-1984 (forestry department, yearly reports) **(22)**
SCA AB, Lövbekämpning 1955-1958 (eradication of deciduous trees) **(23)**
SCA AB, Inventering Lövbekämpning 1956-1961 (inventory, eradication of deciduous trees) **(24)**

Värmlandsarkiv, Karlstad (archive for the Värmland county)
Uddeholms AB
Skogsförvaltningen (forestry management)
Biocidfrågan 28/1970 (the question of biocides) **(25)**

StoraEnso arkiv, Falun
Stora Kopparbergs Bergslags AB, Årsberättelse 1957-1965 (yearly reports) **(26)**
Skogsavdelningen år 1966, reg nr 657.8 (forestry department) **(27)**
Skog Vol. 1741, utförda skogsvårdsåtgärder from 1969-82 (finalized forest management actions) **(28)**
Skog Vol. 1743, utförda skogsvårdsåtgärder from 1983 (finalized forest management actions) **(29)**

S11: Map showing the primary study area, Norrland in dark green with the adjacent counties of Dalarna and Värmland below and east.

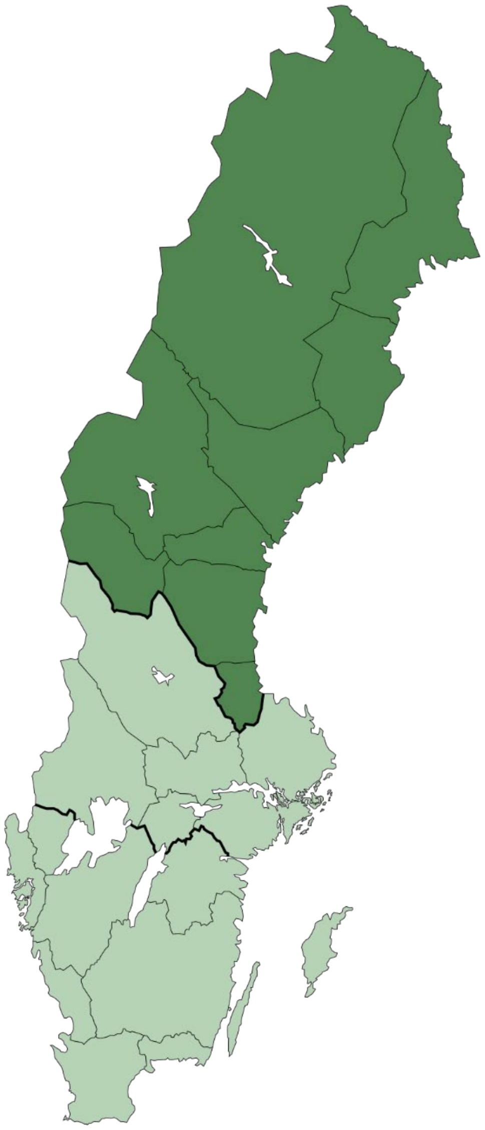

S12: Questions asked in the semi-structured interviews with foresters, forest workers and activists. All interviews conducted in Swedish, and the questions below translated to English by the authors

INTERVIEW QUESTIONS – Forest workers and foresters

What is your name?

Birth place and birth date?

Where have you lived throughout your life?

What has your occupation (s) been?

When and how did you work with herbicides in forestry?

a) Air spraying

b) Spraying manually by hand

c) Other

Injection with herbicides into the trunk of the tree

a) Describe the practise?

b) What tools were used?

c) Why was this done?

d) What tree species were treated? How large were the trees? In what type of forest was the method used?

e) Who had ordered the job?

f) If you planned or supervised this type of job, what was your role?

What information did you have about herbicides when you started working? Who informed you and what did they say about practice and safety?

What was your view on herbicide treatment at the time? Was the method effective? Was it good for the forest?

What was your view on how chemicals were handled at the time? Did you have worries about your health? Were you worried about the effects on humans and animals?

You worked in forestry during the whole herbicide period. What did you think about the debate at the time (nature conservation vs forestry companies)? How much did you know?

Did a debate start locally? If so, who took part in the debate locally?

Did you experience a polarization among people you knew? Were some people for and some against?

What are your thoughts today, looking back at the herbicide period?

(Health risks? Effects on the forest? What are your views on the debate in hindsight?)

Do you know about any areas that were controversial, where there were lots of discussions around herbicide spraying?

Do you know of other people who took part in herbicide spraying? Someone we can interview?

(Are you aware of anyone whose health has been affected by working with herbicides?*)

Do you still have any tools left that were used in herbicide treatment?

Are there things you would like to add and which we have not asked?

INTERVIEW QUESTIONS - environmental activists

What is your name?

Birth place and birth date?

Where have you lived throughout your life?

What has your occupation (s) been?

The protest against herbicide spraying – how did they start in your area? Tell us in your own words how it began and this situation developed.

When did the protests start?

What triggered the protests?

Which forests were to be sprayed and how large areas were planned to be sprayed?

Who was the landowner?

Whose initiative was it to start protesting?

Who took part?

Why did you feel compelled to protest against the spraying?

How did you communicate with the press and other media?

Who was there from the forestry company to negotiate with you?

How did the forestry company act at the meetings, how were these discussions?

What were their arguments for spraying?

When did herbicide spraying start in a larger area around your village?

How and why did you become involved in this question?

Did you join in protests in other locations?

Were you in contact with other local or national protest groups? If so, how did you communicate (telephone, meetings?) Were you inspired by other groups?

How important was the engagement of well known people such as Sara Lidman or Christopher Rappe in the debate?

Are there things you would like to add and which we have not asked?
